# An unexpected dual-emissive luminogen with tunable aggregation-induced emission and enhanced chiroptical property

**DOI:** 10.1038/s41467-022-31281-9

**Published:** 2022-06-21

**Authors:** Xinyu Zhang, Huiqing Liu, Guilin Zhuang, Shangfeng Yang, Pingwu Du

**Affiliations:** 1 Hefei National Research Center for Physical Sciences at the Microscale, Anhui Laboratory of Advanced Photon Science and Technology, CAS Key Laboratory of Materials for Energy Conversion, Department of Materials Science and Engineering, iChEM, University of Science and Technology of China, 96 Jinzhai Road, Hefei, Anhui Province 230026 China; 2 College of Chemical Engineering, Zhejiang University of Technology, 18 Chaowang Road, Hangzhou, Zhejiang Province 310032 China

**Keywords:** Optical materials, Interlocked molecules, Synthetic chemistry methodology

## Abstract

In the literature, organic materials with both aggregation-induced emission (AIE) and aggregation-caused quenching (ACQ) effects that can emit with multiple bands both in the solution and aggregated state are rarely reported. Herein we report a novel chiral dual-emissive bismacrocycle with tunable aggregation-induced emission colors. A facile four-step synthesis strategy is developed to construct this rigid bismacrocycle, (1,4)[8]cycloparaphenylenophane (**SCPP[8]**), which possesses a 1,2,4,5-tetraphenylbenzene core locked by two intersecting polyphenylene-based macrocycles. The luminescent behavior of **SCPP[8]** shows the unique characteristics of both ACQ effect and AIE effect, inducing remarkable redshift emission with near white-light emission. **SCPP[8]** is configurationally stable and possesses a novel shape-persistent bismacrocycle scaffold with a high strain energy. In addition, **SCPP[8]** displays enhanced circularly polarized luminescence properties due to AIE effect.

## Abstract (web summary)

Organic materials with both aggregation induced emission (AIE) and aggregation-caused quenching (ACQ) effects that can emit with multiple wavelengths in the solution and aggregated state are rarely reported. Here, the authors report a chiral dual-emissive bismacrocycle which shows the unique ACQ and AIE effects inducing redshift emission with near white-light emission.

## Introduction

Aggregation-induced emission (AIE) phenomenon has enormous potential applications in biological probes, chemical sensors, and optoelectronic materials^1–3^. The concept of AIE has received much attention since Tang et al. described it in 2001 and great progress has been made since then^4–9^. In many traditional systems, luminophores often emit strongly in their dilute solutions but meet with varying degrees of aggregation-caused quenching (ACQ) effect when they are aggregated or clustered. In contrast, non-emissive luminogens are induced to emit by the aggregate formation in the AIE systems^6^. Therefore, AIE luminogens (AIEgens) show remarkable advantages over typical ACQ molecules, especially in their aggregate and solid states^10,11^. However, in the literature, materials with both AIE and ACQ effects that can emit both in the solution and aggregated state are rarely reported. The solution and solid dual-emissive luminogens can exactly fill the gap between ACQ and AIE materials, providing many interesting properties such as stimuli-responsive fluorescence and white-light emission^12–16^. Most of the AIE systems have been identified on the basis of intensity turn-on at a specific wavelength. If AIE sensors have tunable emission colors based on the manipulation of aggregation, they can endow a dimension with the discriminative power and allow naked-eye visualization^17^. Therefore, the development of novel dual-emissive luminogens is highly desired.

**Fig. 1:**
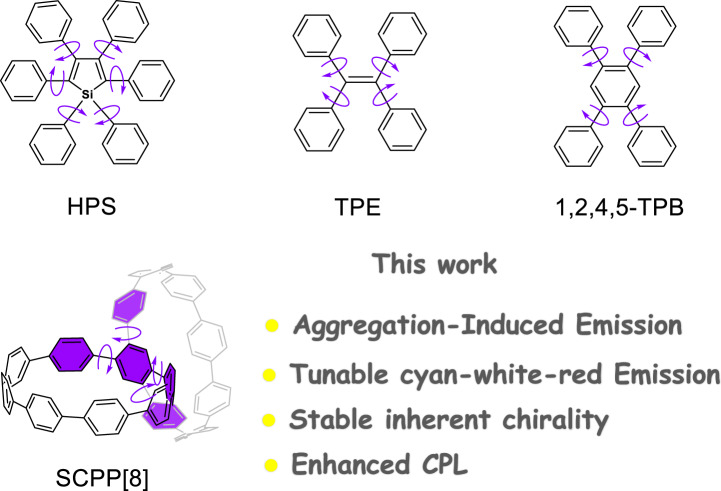
Schematic representation. Top: Typical examples of structural motifs of AIEgens. Bottom: Structure of **SCPP[8]** molecule.

Typical AIEgens, such as hexaphenylsilole (HPS) and tetraphenylethene (TPE), have been well studied (Fig. 1)^18–20^. In solution, the dynamic rotations of the phenyl rings in TPE as a relaxation channel for the excitons to dissipate. Upon aggregate formation, such motion is restricted due to the restricted intramolecular rotation (RIR) and intermolecular π-π stacking interaction derived from the highly twisted molecular conformation^21,22^. By immobilization of propeller-shaped AIE molecules with various functional groups, new AIEgens will form and exhibit many different properties^23–25^. This design approach also takes advantage of the emission features in the AIE-active core, providing new systems with novel physical properties and applications^12,26,27^. Moreover, when attaching chiral moieties to the propeller-shaped structures, many new applications are reported in chiral sensors and chiroptical materials^28–31^. 1,2,4,5-tetraphenylbenzene (1,2,4,5-TPB) is another kind of AIEgens that also features a propeller-shaped structure^32^ (Fig. 1), which is an attractive building block for the construction of chiral molecules with novel AIE properties. In sharp contrast to linear *para*-phenylenes, cycloparaphenylenes (CPPs) are radial π-conjugated macrocycles that compose of *para*-connected benzene rings with unique properties^33–36^. Dimeric structures with two CPP-based macrocycles have attracted considerable research due to their interesting structures, novel physical properties, and potential applications^12,37–48^. However, there is no report of chiral bismacrocycle with AIE effect. By bridging two CPP-based macrocycles in a central benzene, a chiral bismacrocycle molecule with AIE-active 1,2,4,5-TPB core can be formed. Although we recently synthesized a [10]CPP-based bismacrocycle (**SCPP[10]**)^48^, its poor solubility and large ring size prevent us to resolve the enantiomers and study the chiral properties. The synthesis of smaller bismacrocycle is attractive due to better solubility for enantiomeric resolution and excellent size-dependent properties for AIE study.

Herein, by immobilization of the AIE-active 1,2,4,5-TPB with rigid *para*-phenylene units, a novel inherently chiral and configurationally stable bismacrocycle was synthesized via a facile four-step synthesis. **SCPP[8]** can emit efficiently both in solution (cyan) and in aggregation (red). The fluorescence emission maximum of **SCPP[8]** in the aggregated state showed a remarkable redshift (102 nm) from that in the dilute solution. More intriguingly, multicolor cyan-white-red photoluminescence can be tuned by manipulating the aggregation, realizing near white-light emission with a CIE coordinate at about (0.33, 0.37). **SCPP[8]** possesses a novel scaffold of nonracemizable chiral structure with enhanced chiroptical properties due to AIE effect. The physical, (chir)optical, and inherently chiral properties were investigated using different techniques.

## Results

### Synthesis and characterizations of SCPP[8]

**Fig. 2:**
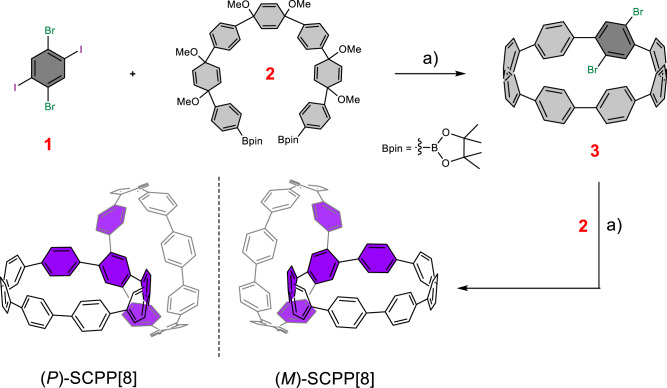
Design and synthesis procedure for inherently chiral SCPP[8] with 1,2,4,5-TPB core. Reagents and conditions: **a** (i) Pd(PPh_3_)_4_, KOH, THF/H_2_O, 2 days at 73 °C; (ii) H_2_SnCl_4_, THF, RT.

The synthesis of a small chiral bismacrocycle with a 1,2,4,5-TPB core poses a great challenge, as a high strain is introduced into the curved molecule. Despite many attempts, our previous approach^48^ using an -OTf functional group was unsuccessful for the synthesis of **SCPP[8]**. Next, we chose a 1,2,4,5-halogen-substituted benzene to react with a C-shaped synthon by a Suzuki coupling reaction to produce a macrocyclic precursor, followed by reductive aromatization to generate a bisubstituted macrocycle (Fig. 2). Since the bisubstituted benzene can rotate rapidly, the enantiomers cannot be separated. Under a similar coupling reaction, the bisubstituted CPP macrocycle was further reacted with the C-shaped synthon to lock the central benzene and fix its rotation, resulting in an inherently chiral bismacrocycle with a 1,2,4,5-TPB core locked by rigid polyphenylene units.

The successful synthesis of **SCPP[8]** can be supported by different characterization techniques, including high-resolution mass spectrometry (HR-MS), ^1^H NMR, ^13^C NMR, 2D ^1^H-^1^H COSY NMR, 2D NOESY NMR, 2D (H, C)-HSQC NMR, 2D (H, C)-HMBC NMR, and variable-temperature ^1^H NMR (Supplementary Figs. 1–16). The molecular weight of **SCPP[8]** was determined by MALDI-TOF MS spectrometry. The main peak at *m*/*z* 1138.4593 was observed (calculated for C_90_H_58_ [*M*]^+^: 1138.4538), which matched well with the calculated data. Variable-temperature ^1^H NMR studies from 25 to 125 °C do not show significant splitting of the resonances in the ^1^H NMR spectra, suggesting the good conformational stability of **SCPP[8] (**Supplementary Fig. 16). Besides, the crystalline solid can be obtained by slow diffusion of methanol into a **SCPP[8]** solution in dichloromethane (see below). Unfortunately, these crystals are extremely fragile, and the large crystals tend to break immediately once exposed to air. Therefore, so far the crystal structure of **SCPP[8]** cannot be resolved.

### Theoretical Calculations of SCPP[8]

**Fig. 3:**
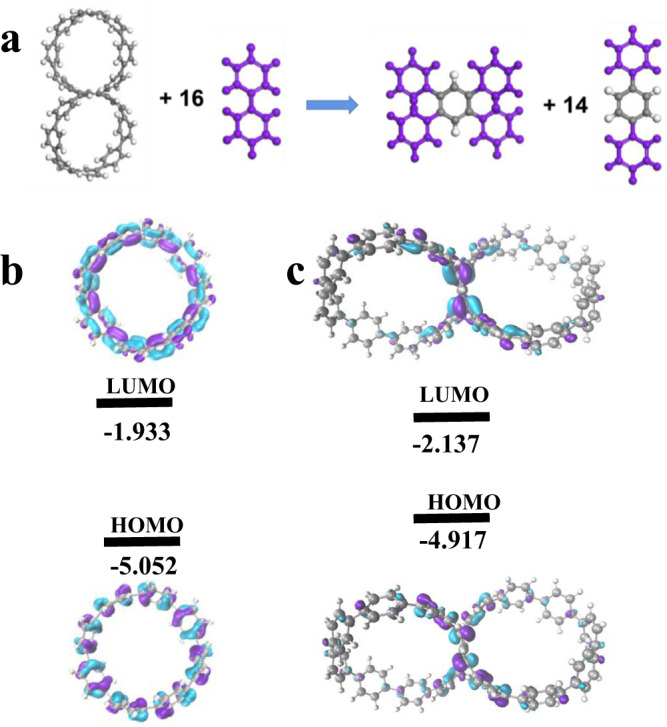
Theoretical calculations. **a** Homodesmotic equation for the calculation of strain energy for **SCPP[8]**. **b** LUMO and HOMO of [8]CPP. **c** LUMO and HOMO of **SCPP[8]**.

**SCPP[8]** has a 127 kcal/mol of strain energy which equates to approximately 8.47 kcal/mol per benzene ring (Fig. 3a and Supplementary Table 2), which is close to the strain of 9.25 kcal/mol per benzene ring in CPP[8]^49^. We concluded that the structural factors contribute to the high strain energy in **SCPP[8]**. Compared with the individual phenylene, the central benzene bridged by two carbon nanorings has a much larger resistance to twisting. In addition, the limited conformational freedom in **SCPP[8]** leads to significant multiple repulsive interactions between adjacent phenylene fragments. The geometrical optimization results indicated that **SCPP[8]** features chiral *C*_2_ symmetry, and the twist angle in the bridging phenyl is ~19.55^o^. The frontier molecular orbitals of [8]CPP and **SCPP[8]** are shown in Figs. 3b, c, respectively. The energy levels of the occupied molecular orbitals of **SCPP[8]** move to higher positions than those of [8]CPP, while the unoccupied molecular orbitals move to lower positions than those of [8]CPP. Therefore, the **SCPP[8]** achieves a smaller HOMO-LUMO gap of ~2.780 eV compared to [8]CPP (Supplementary Tables 3 and 5–6).

### Photophysical properties of SCPP[8]

**Fig. 4:**
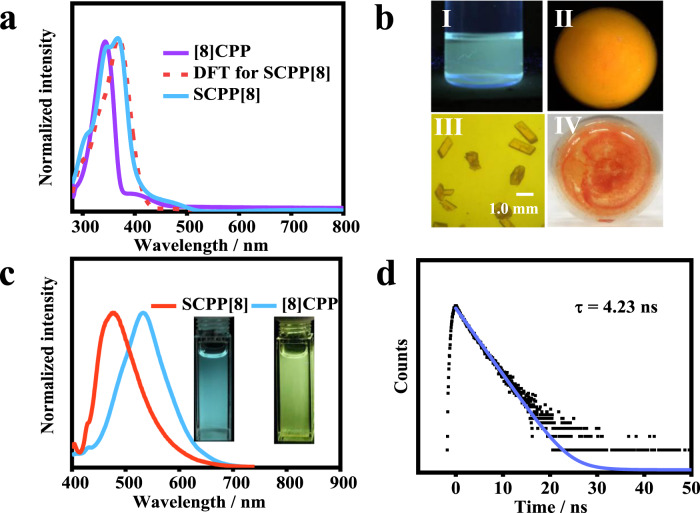
Photophysical properties of SCPP[8]. **a** UV–Vis absorption spectra of **SCPP[8]** (blue) and [8]CPP (purple) in THF. Simulated curve of UV spectrum for **SCPP[8]** (dotted line). **b** Photos of **SCPP[8]** in THF solution (I), as powder (II) under 365 nm UV light, as crystal (scale bar: 1.0 mm) (III), and as powder (IV) under daylight. **c** Fluorescence spectra of **SCPP[8]** (red) and [8]CPP (blue) in THF. Insert: [8]CPP and **SCPP[8]** in THF solution under 365 nm UV light. **d** Emission lifetime for **SCPP[8]**.

The photophysical properties of **SCPP[8]** were further studied by UV–Vis absorption spectroscopy, fluorescence spectroscopy, and time-resolved fluorescence decay (Fig. 4). A single macrocyclic [8]CPP was used as a reference compound for comparison. The lower symmetry of **SCPP[8]** (*C*_2_) in comparison with the circular CPPs (*D*_2_) results in a more complex pattern of electronic transitions, as determined in time-dependent density functional theory (TD-DFT) calculations. The absorption band of **SCPP[8]** was in the region between 265 and 436 nm, and the maximum absorption peak (*λ*_max_) appears at 368 nm (Fig. 4a). The extinction coefficients reach up to 1.22 × 10^5^ M^−1^ cm^−1^. To identify the difference of optical properties, density functional theory calculations were performed by using Gaussian 16 software. In accordance with the aforementioned decrease in the HOMO-LUMO gap, the absorption spectrum of **SCPP[8]** showed an obvious redshift compared with [8]CPP. Based on the calculated results, an excellent correlation between the calculated and experimental absorption spectra was observed. The three main absorption peaks can be attributed to the transition of HOMO-2 → LUMO for the peak at 368 nm, HOMO → LUMO+2 for the peak at 346 nm, and HOMO-1 → LUMO+5 for the peak at 305 nm. In addition, the observed absorption peak of [8]CPP at 340 nm can be assigned to the transition of HOMO-1 → LUMO and HOMO → LUMO+1 (Supplementary Figs. 22, 23, and Table 4). The redshift effect of the absorption spectrum in **SCPP[8]** compared to [8]CPP was ascribed to the shift of the frontier molecular orbitals.

The steady-state fluorescence emission spectrum of **SCPP[8]** was further measured (Fig. 4c). Emission band was observed under excitation at 380 nm. **SCPP[8]** and [8]CPP showed cyan and yellow fluorescence in tetrahydrofuran (THF) with fluorescence maxima at 475 nm and 533 nm, respectively. The fluorescence quantum yield (*Φ*_F_) was determined to be ~3% using anthracene in ethanol as the reference (*Φ*_F_ = 30%), and the yield is lower than that of [8]CPP (*Φ*_F_ = 10%), which probably results from its high proportion of nonradiative decay in the low-energy emission region.

The fluorescence lifetime (*τ*_s_) of **SCPP[8]** in THF was measured by time-resolved fluorescence decay using the time-resolved photoluminescence (TRPL) technique. The emission lifetime of **SCPP[8]** was determined to be *τ* = 4.23 ns at 475 nm by mono-exponential decay fitting when excited at 390 nm (Fig. 4d).

### AIE properties and tunable emission of SCPP[8]

**Fig. 5:**
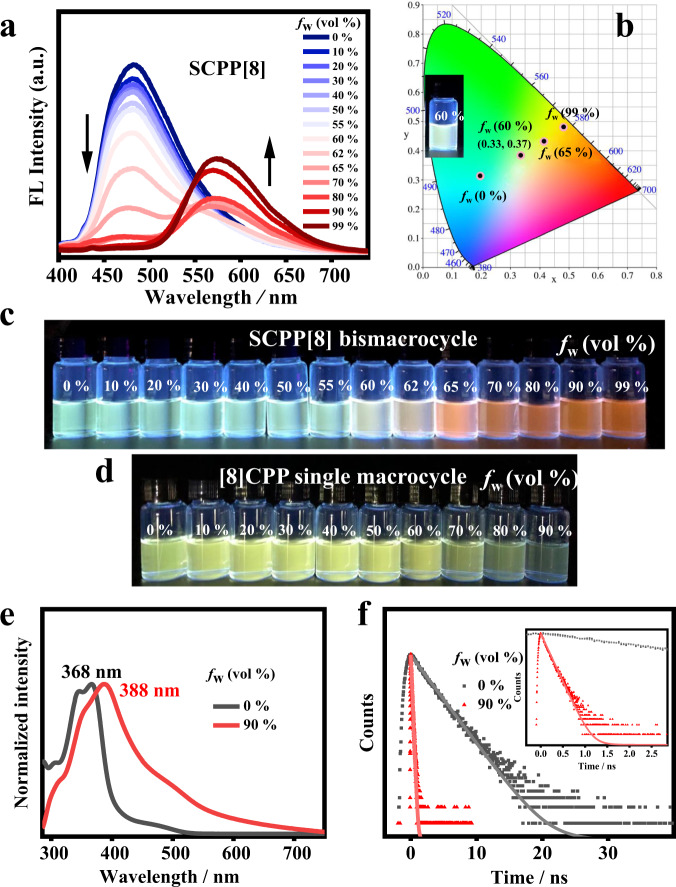
AIE properties and tunable emission. **a** Fluorescence spectra of **SCPP[8]** in THF/H_2_O mixtures with different volume fractions of H_2_O (*f*_w_). **b** CIE 1931 chromaticity diagram of **SCPP[8]** in THF/H_2_O mixtures. Insert: The near white-light of **SCPP[8]** in aqueous THF with *f*_w_ = 60 vol% under 365 nm UV light. **c** Emission color changes of **SCPP[8]** from cyan to red in aqueous THF with *f*_w_ = 0-99 vol% under 365 nm UV light. **d** Emission change of [8]CPP in aqueous THF with *f*_w_ = 0-90 vol% under 365 nm UV light. **e** The UV–Vis absorption spectra of **SCPP[8]** in THF solution and in 90:10 H_2_O/THF (*v*/*v*) solution. **f** Emission lifetimes of **SCPP[8]** in THF solution and in 90:10 H_2_O/THF solution. Insert: Emission lifetime of **SCPP[8]** in aggregation.

Traditional organic luminescent materials can emit efficiently either in dilute solution or in the aggregated state. However, an interesting phenomenon was found when we studied the luminescence behavior of **SCPP[8]**. Upon excitation with 365 nm UV light, **SCPP[8]** in THF solution emitted cyan fluorescence, while the powder sample exhibited an orange fluorescence. The powder and crystal of **SCPP[8]** were orange under daylight (Fig. 4b). All these observations indicated that **SCPP[8]** has different emission properties upon aggregation with a possible AIE effect. The luminescent properties of the **SCPP[8]** were further investigated in H_2_O/THF mixtures with different water fractions (*f*_w_), in which THF is a good solvent and water is a poor solvent. The fluorescence intensity of **SCPP[8]** maximized at ~475 nm decreased as the water fraction was increased, showing an obvious quenching effect due to aggregation. An interesting phenomenon was observed at *f*_w_ > 60 vol%. A new emission band appeared at ~577 nm and the fluorescence intensity was further enhanced when a higher content of water was added, which could be ascribed to the obvious AIE effect (Fig. 5a). Meanwhile, the maximum emission wavelength was redshifted by 102 nm, from 475 to 577 nm. Further characterizations of **SCPP[8]** at *f*_w_ = 90 vol% were obtained by UV–Vis absorption spectroscopy and time-resolved fluorescence decay technique. The absorption spectrum of **SCPP[8]** displays a much broader and red-shifted band compared with its absorption spectrum in THF solution (Fig. 5e), presumably originating from the strong π-π stacking interactions between individual molecules in aggregation. The PL decay is much faster in aggregation than that in solution and the decay dynamics are fitted by a double-exponential function (Fig. 5f). A short-lived species with a lifetime of 1.81 ns (92.96 %) and a long-lived species with a lifetime of 30.94 ns (7.04%) for emission monitored at 577 nm, which probably indicates that two relaxation pathways are involved in the decay process. For comparison, when [8]CPP was dissolved in THF, the dilute solution showed strong yellow luminescence. With the gradual increase in the fraction of water, the emission became weakened and no any AIE effect was observed. At *f*_w_ = 80 vol% and *f*_w_ = 90 vol%, the emission was significantly quenched due to severe aggregate formation, indicating a typical ACQ effect (Supplementary Fig. 17 and Fig. 5d). For comparison, the emissive properties of **SCPP[10]** with larger rings were also investigated in H_2_O/THF mixtures (Supplementary Fig. 18). However, **SCPP[10]** only showed some AIE effect with no dual-emissive property and no significant redshift in emission peaks.

The emission color changes of **SCPP[8]** from cyan to red were recorded in aqueous THF with *f*_w_ = 0–99 vol% under 365 nm UV-light (Fig. 5c). Intriguingly, a near white-light emission was observed at *f*_w_ = 60 vol%. In the literature, most organic white-light emitters depend on the combination of several components that emit different colors of light^50–52^. Compared with multicomponent emitters, single-molecule systems with white-light emission have excellent stability, reproducibility, and a simplified fabrication process^53–55^. The photoluminescence spectra of **SCPP[8]** at *f*_w_ = 0–99 vol% were obtained, and their corresponding luminescent color coordinates were calculated and plotted in the CIE 1931 chromaticity diagram (Fig. 5b). The near white-light emission has a CIE coordinate of about (0.33, 0.37). Therefore, **SCPP[8]** can be considered as a potential candidate for organic white-light-emitting materials.

The present fluorescence behaviors are quite different from those of traditional ACQ or AIE materials. **SCPP[8]** is an unexpected dual-emissive luminogen that can emit both in the solution and aggregated state. The luminescence behavior of **SCPP[8]** combines the characteristics of the ACQ effect and AIE effect, inducing tunable emission including near white-light emission. In contrast, the reference compound [8]CPP only shows a typical ACQ effect. Therefore, the unique photophysical properties in **SCPP[8]** can be attributed to the locked conjugated bismacrocycle structure and the AIE-active 1,2,4,5-TPB core.

### Chirality and enhanced CPL

**Fig. 6:**
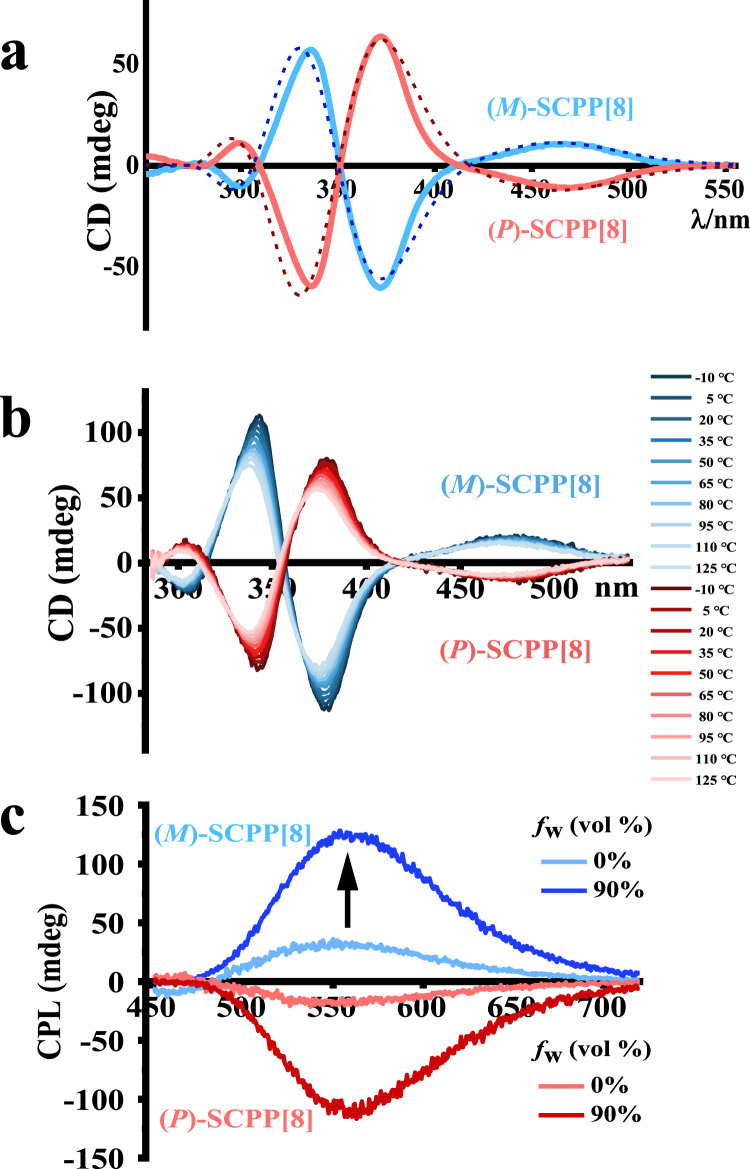
Chirality and enhanced CPL. **a** Experimental and theoretical CD spectra for **(*P*)-SCPP[8]** and **(*M*)-SCPP[8]**. The solid lines are the experimental spectra of **(*M*)-SCPP[8]** (blue) and **(*P*)-SCPP[8]** (red) in dichloromethane. The dashed lines are theoretical spectra of **(*M*)-SCPP[8]** (dark blue) and **(*P*)-SCPP[8]** (dark red) from TD-DFT calculations. **b** Temperature-dependent CD spectra of **(*M*)-SCPP[8]** (blue) and **(*P*)-SCPP[8]** (red) from −10 to 125 °C at 15 °C intervals. **c** CPL spectra of **(*M*)-SCPP[8]** (blue) and **(*P*)-SCPP[8]** (red) in THF solution and in 90:10 H_2_O/THF solution. Excited at 355 nm.

The structure of **SCPP[8]** consists of a twisted benzene ring bridged by polyphenylene units, resulting in a stable and rigid conformation. This excellent conformational stability enables complete enantiomeric resolution and characterizations. After purification by flash column chromatography, the resolution of racemic samples was achieved by a recycling HPLC system. After only two cycles, we succeeded in the isolation of the complete set of two enantiomers **(*P*)-SCPP[8]** and **(*M*)-SCPP[8]**. The extremely narrow peaks in HPLC strongly indicated the excellent separation of the two enantiomers, and the molar ratio of the two isomers was determined to be approximately 1:1 (Supplementary Fig. 19). The enantiomeric relationship of the **(*P*)-SCPP[8]** and **(*M*)-SCPP[8]** was identified using circular dichroism (CD) spectroscopy. In Fig. 6a, the CD spectrum of **(*M*)-SCPP[8]** showed two negative Cotton effects at 299 and 371 nm and two positive ones at 336 and 467 nm. Being the enantiomer of **(*M*)-SCPP[8]**, **(*P*)-SCPP[8]** gave the expected mirror-imaged CD spectrum, confirming their enantiomeric relationship. The magnitude of the CD spectra can be quantified by the dissymmetry factor (*g*_CD_), which is the ratio of CD intensity to the corresponding absorption. A large |*g*_CD_| of ~0.012 was obtained for the enantiomers of **SCPP[8]**. To further elucidate the absolute configuration of **(*P*)-SCPP[8]** and **(*M*)-SCPP[8]**, we investigated the theoretical CD spectra on the basis of TD-DFT calculations, as shown in Fig. 6a (dashed plot). The theoretical CD spectra of the resolved enantiomers were in good agreement with the experimental results.

To gain insight into the configurational stability of **(*P*)-SCPP[8]** and **(*M*)-SCPP[8]**, we used temperature-dependent CD spectra to investigate the conformational behavior of these two enantiomers. Chlorobenzene was chosen as the solvent as it allowed access to a wide range of temperatures. To investigate the temperature dependence of the CD-signals, the samples were heated from −10 to 125 °C, and CD spectra were recorded at 15 °C intervals (Fig. 6b). The results clearly showed that optical activity of **(*P*)-SCPP[8]** and **(*M*)-SCPP[8]** have no significant change even after heating to 125 °C. The slight change can be attributed to the influence of the solvent at different temperatures, and the change can be recovered by cooling down the solvent (Supplementary Fig. 20). This result showed that the resolved enantiomers of **SCPP[8]** are configurationally stable, maintaining their optical activity in a wide range of temperatures. These rigid bismacrocycles contain nonracemizable structures, and there is no pathway for racemization without breaking a C–C bond between phenylenes. The enantiomeric purity of **SCPP[8]** can be remained due to its unique structural geometry, suggesting good enantiomeric stability for further potential applications. Therefore, the isolation and characterization of **SCPP[8]** enantiomers shed light on a novel scaffold of nonracemizable chiral bismacrocycles with potential applications as chiroptical material.

The circularly polarized luminescence (CPL) is quantified through the luminescence dissymmetry factor (*g*_lum_), which is defined as the ratio of the difference of the left- and right-circularly polarized light to half of the total luminescence^56^. CPL-active materials fabricated from AIEgens show much larger dissymmetry factors in the solid state than the molecules in solution^1,23,57^. Significantly, **(*M*)-SCPP[8]** and **(*P*)-SCPP[8]** exhibited highly enhanced CPL signals in the aggregated state (Fig. 6c). In a THF solution, the *g*_lum_ was 6.9 × 10^−3^ for **(*M*)-SCPP[8]** at 554 nm and −6.1 × 10^−3^ for **(*P*)-SCPP[8]** at 558 nm. In a H_2_O/THF (90:10, *v*/*v*) solution, the |*g*_lum_ | is ~3-fold larger than that in solution, which is 1.9 × 10^−2^ for **(*M*)-SCPP[8]** at 562 nm and −1.8 × 10^−2^ for **(*P*)-SCPP[8]** at 564 nm. The results showed **SCPP[8]** has a relatively high luminescence dissymmetry factor compared with many chiral small organic molecules (SOMs) with AIE properties and CPL-active bismarocycles^23,42,43,58–64^ (Supplementary Fig. 21 and Table 1). Most SOMs exhibit low |*g*_lum_| values (10^−5^−10^−3^)^56,65^. In addition, the CPL features of most SOMs are deteriorated in the solid state due to the ACQ effect. Therefore, chiral **SCPP[8]** with AIE effect has promising potential in applications as a novel CPL-active materials.

## Discussion

In summary, we report the construction of a dual-emissive **SCPP[8]** bismacrocycle by immobilization of 1,2,4,5-TPB core with two rigid macrocycles. The luminescence behavior of **SCPP[8]** combines the characteristics of the ACQ effect and AIE effect, inducing tunable emission from cyan to red including near white-light emission. The enantiomers of **(*M*)-SCPP[8]** and **(*P*)-SCPP[8]** are separated by a recycling chiral HPLC, and the high configurational stability of two enantiomers are demonstrated by temperature-dependent CD spectra. Further, **(*M*)-SCPP[8]** and **(*P*)-SCPP[8]** exhibit highly enhanced CPL properties due to AIE effect. **(*M*)/(*P*)-SCPP[8]** will have potential applications in AIE sensors, white-light emitters, and chiral materials.

## Methods

### Synthesis of compound 1

To a mixture of 1,4-dibromobenzene (5 g, 21.19 mmol) and iodine (21 g, 82.74 mmol) was added concentrated sulfuric acid (50 ml). Thereafter, the solution was heated at 130 °C for 24 h. Upon cooling to room temperature, 20 mL CHCl_3_ was added and the suspension was washed with 10% aqueous sodium bisulfite, sodium bicarbonate, and H_2_O. The organic solvent was then removed and a white solid (7.75 g, 75%) was obtained. ^1^H NMR (CDCl_3_, 400 MHz): δ (ppm) 8.19 (s, 2H). ^13^C NMR (CDCl_3_, 100 MHz): δ (ppm) 141.54, 128.04, 99.46. The characterization matches well with the reported data^66^.

### Synthesis of compound 2

The synthesis of compound **2** was carried out using a modified procedure^67^: 1,1’-(1,4-dimethoxy-2,5-cyclohexadiene-1,4-diyl)bis[4-[4-(4-bromophenyl)−1,4-dimethoxy-2,5-cyclohexadien-1-yl]benzene] (1.0 g, 1.14 mmol) was dissolved in THF (15 ml) and cooled to −78 °C. To this solution was added a 2.5 M solution of *n*-BuLi in hexanes (1.0 mL, 2.50 mmol) over 2 min. Then, 2-isopropoxy-4,4,5,5-tetramethyl-1,3,2dioxaborolane (0.6 mL, 2.89 mmol) was added rapidly and the solution was stirred for 20 min. Water (10 mL) was added to the solution and the mixture was allowed to stir for 15 min at room temperature. The aqueous layer was extracted with CH_2_Cl_2_ and concentrated under reduced pressure. The crude product was purified by column chromatography (hexane/ethyl acetate = 1/1, *v*/*v*) to afford compound **2** (0.9 g, 81% yield) as a white solid. ^1^H NMR (CDCl_3_, 400 MHz): δ (ppm) 7.76 (d, *J* = 8.3 Hz, 4H), 7.53 (dd, *J* = 11.0 Hz; *J* = 8.5 Hz, 2H), 7.40 (d, *J* = 8.3 Hz, 4H), 7.34 (s, 6H), 6.09 (dt, *J* = 5.9 Hz; *J* = 3.3 Hz, 12H), 3.42 (dd, *J* = 4.6 Hz; *J* = 2.6 Hz, 15H), 3.28 − 3.24 (m, 3H), 1.33 (s, *J* = 2.5 Hz, 24H). ^13^C NMR (CDCl_3_, 100 MHz): δ (ppm) 146.37, 142.65, 142.51, 134.81, 133.90, 133.57, 133.29, 133.19, 133.03, 128.26, 125.91, 125.17, 83.62, 74.74, 74.49, 74.45, 74.21, 51.84, 24.74. HR-MS (MALDI-TOF) *m/z* calcd. for C_60_H_70_B_2_O_10_Na [M + Na]^+^: 995.5053, found: 995.6781. The characterization matches well with the reported data^67^.

### Synthesis of compound 3

To a solution of compound **1** (130 mg, 0.27 mmol), compound **2** (260 mg, 0.27 mmol), KOH (185 mg, 3.30 mmol), THF (250 mL) and H_2_O (10 mL) was added Pd(PPh_3_)_4_ (35 mg, 0.03 mmol) under an argon atmosphere. The resulting solution was stirred at 73 °C for 48 h and then cooled to room temperature. The solvent was removed under reduced pressure, and the remaining aqueous fraction was extracted with CH_2_Cl_2_, and dried over anhydrous MgSO_4_. The solution was concentrated under reduced pressure to afford the crude product as a yellow solid for the next step without further purification.

To a mixture of SnCl_2_·2H_2_O (365 mg, 1.60 mmol) and dry THF (30 mL) was added HCl (0.25 mL, 12 mol/L) under an argon atmosphere and the resulting mixture was stirred at room temperature for 30 min. Then, the above stannic acid solution was added dropwise to the above crude product with a syringe under an argon atmosphere and stirred overnight at room temperature. After that, aqueous sodium hydroxide was added to quench the reaction and the solvent was removed under reduced pressure. Then, the remaining aqueous fraction was extracted with CH_2_Cl_2_, dried over anhydrous MgSO_4_ and was purified by chromatography on a silica gel column with hexane/CH_2_Cl_2_ as the eluent (*v*/*v*, 4:1) to give compound **3** as a yellow solid (30 mg, 15% over two steps). ^1^H NMR (CDCl_3_, 600 MHz): δ (ppm) 7.54 (d, *J* = 8.8 Hz, 4H), 7.51 (d, *J* = 8.8 Hz, 4H), 7.46 (s, 2H), 7.44 (d, *J* = 4.0 Hz, 8H), 7.44-7.43 (s, 2H), 7.41 (d, *J* = 2.1 Hz, 8H), 7.40 (s, 2H). ^13^C NMR (CDCl_3_, 150 MHz): δ (ppm) 141.03, 140.49, 138.44, 138.10, 138.08, 138.03, 137.63, 136.57, 136.44, 130.68, 128.27, 127.71, 127.55, 127.44, 121.02. HR-MS (MALDI-TOF) *m/z* calcd. for C_48_H_30_Br_2_ [M]^+^: 766.0781, found: 766.0798.

### Synthesis of SCPP[8]

To a mixture of **3** (55 mg, 72 μmol), **2** (70 mg, 72 μmol) and KOH (55 mg, 0.48 mmol) in a round-bottomed flask (500 mL) were added THF (150 mL), H_2_O (30 mL), and Pd(PPh_3_)_4_ (19 mg, 17 μmol) under an argon atmosphere. Thereafter, the solution was heated at 73 °C for 48 h. After cooling to room temperature, the solvent was removed under vacuum and the remaining aqueous fraction was extracted with CH_2_Cl_2_. The combined organic layer was dried over anhydrous MgSO_4_ and concentrated under reduced pressure to afford the crude product as a yellow solid that was used in the next step without further purification.

To a 50 mL round-bottom flask containing a magnetic stirring bar were added SnCl_2_·2H_2_O (205 mg, 0.91 mmol), THF (20 mL) and concentrated HCl/H_2_O (0.20 mL, 12 mol/L) were added, and the resulting mixture was further stirred at room temperature for 30 min. The solution of H_2_SnCl_4_/THF was added dropwise to a solution containing the above crude product in 5 mL of THF. After stirring the mixture at room temperature for 2 h, the mixture was quenched with aqueous sodium hydroxide, extracted with CH_2_Cl_2_, dried over Na_2_SO_4_, and concentrated under reduced pressure. The crude product was purified by column chromatography (hexane/CH_2_Cl_2_ = 3/1, *v*/*v*) to afford **SCPP[8]** (16 mg, 22% over two steps) as an orange solid. ^1^H NMR (CDCl_3_, 600 MHz): δ (ppm) 7.79 (d, *J* = 9.0 Hz, 8H), 7.70 (s, 2H), 7.55 (s, 8H), 7.52 (s, 16H), 7.50 (d, *J* = 8.7 Hz, 8H), 7.41 (d, *J* = 8.2 Hz, 8H), 7.38 (d, *J* = 6.1 Hz, 8H). ^13^C NMR (CDCl_3_, 150 MHz): δ (ppm) 138.44, 138.18, 137.97, 137.81, 137.66, 137.55, 137.38, 134.62, 127.61, 127.57, 127.29, 127.18. HR-MS (MALDI-TOF) *m/z* calcd. for C_90_H_58_ [M]^+^: 1138.4538, found: 1138.4593.

### Spectroscopy analysis

UV–Vis spectra were obtained on a WFZ UV-3802 scanning quasi-double beam ultraviolet-visible spectrophotometer (UNICO, Shanghai, China) equipped with environmental tritium-powered lighting system (Japan) in quartz cuvettes. Steady-state fluorescence spectra were obtained with a Horiba FluoroMax-4 compact spectrofluorometer equipped with an ozone-free xenon lamp optics source. The spectra were uncorrected and quartz cells (1 cm) were used for all spectroscopic measurements. The PL lifetimes were measured by time-resolved fluorescence decay equipped with TBX picosecond photon detection module (HORIBA Scientific).

### HPLC experiments

The enantiomers were separated by reserve-phase high-pressure liquid chromatography using a longer cholesterylated silica gel column (COSMOSIL Cholester column, 10 Φ × 250 mm, NACALAI TESQUE, INC.). The separation was done by a recyclable HPLC (JAI LC-9104 recycling preparative HPLC) system equipped with a UV–Vis detector (JAI UV detector 310; 350 nm). Chromatography condition: toluene/acetonitrile = 1/4 (*v*/*v*), 4.0 mL/min, 40 °C.

### CD study

CD experiments were performed on a Jasco J-1500 circular dichroism spectrometer equipped with a PFD-425S/15 Peltier-type temperature controller. A 1.0 cm quartz cuvette was used. The scanning speed was 100 nm/min and D.I.T. value was 1.0 s. The flow rate of nitrogen was 5 L/min. The value of *g*_abs_ was calculated using the data mode of *g*_CD_ = CD(mdeg)/(32980*Abs), where the “CD” and the “Abs” can be directly obtained from CD spectra.

### CPL measurements

A JASCO CPL-300 spectrometer was used for the CPL experiments. The error range, ±5%. The value of luminescence dissymmetry factor (*g*_lum_) was calculated using the formula of *g*_lum_ = 2[ellipticity/(32980/ln10)]/total fluorescence intensity at the CPL extremum, where the “ellipticity” and the “total fluorescence intensity” can be directly obtained from CPL spectra.

## Supplementary information

Supplementary Information

## Data Availability

Materials and methods, experimental procedures, useful information, characterization studies, ^1^H NMR spectra, ^13^C NMR spectra, and mass spectrometry data are available in the Supplementary Information. Additional data that support the findings of this study are available from the corresponding author upon request.
