# Supplementary material for: An unexpected dual-emissive luminogen with tunable aggregation-induced emission and enhanced chiroptical property
Source: Nat Commun. 2022 Jun 21;13:3543. doi: 10.1038/s41467-022-31281-9 (PMC9213505; doi:10.1038/s41467-022-31281-9)
Supplement: Supplementary file 1 — Supplementary Information [file 41467_2022_31281_MOESM1_ESM.pdf]

Supplementary Information

An unexpected dual-emissive luminogen with tunable aggregation-induced emission and enhanced chiroptical property

Xinyu Zhang,[1] Huiqing Liu,[1] Guilin Zhuang,[2] Shangfeng Yang,[1]* Pingwu Du[1]*

[1] Hefei National Research Center for Physical Sciences at the Microscale, Anhui Laboratory of Advanced Photon Science and Technology, CAS Key Laboratory of Materials for Energy Conversion, Department of Materials Science and Engineering, iChEM, University of Science and Technology of China, 96 Jinzhai Road, Hefei, Anhui Province, 230026, China

[2] College of Chemical Engineering, Zhejiang University of Technology, 18 Chaowang Road, Hangzhou, Zhejiang Province, 310032, China

*Corresponding author: dupingwu@ustc.edu.cn; sfyang@ustc.edu.cn
Tel/Fax: 86-551-63606207

Supplementary Notes

NMR spectra were recorded on a Bruker BioSpin (^1H 400 MHz, ^{13}C 100 MHz) spectrometer or a JEOL JNM-ECZ600R (^1H 600 MHz, ^{13}C 150 MHz) NMR spectrometer. Chemical shifts were reported as the delta scale in ppm relative to tetramethylsilane (δ = 0.00 ppm), $CHCl_3$ (δ = 7.26 ppm) for ^1H NMR and $CDCl_3$ (δ = 77.0 ppm) for ^{13}C NMR. Data were reported as follows: chemical shift, multiplicity (s = singlet, d = doublet, t = triplet, m = multiplet), coupling constant (Hz), and integration. High resolution mass spectrometry (HR-MS) analyses were carried out using MALDI-TOF-MS techniques. UV-Vis absorption spectra were performed on a UNIC-3802 spectrophotometer. All solvents for syntheses were dried by distillation under nitrogen prior to use (tetrahydrofuran and 1,4-dioxane were distilled after reflux with sodium under nitrogen). Other chemicals were obtained from commercial suppliers (Innochem or Acros). Air-sensitive reactions were all carried out under argon or nitrogen.

Supplementary Methods

Physical Characterizations of Compounds

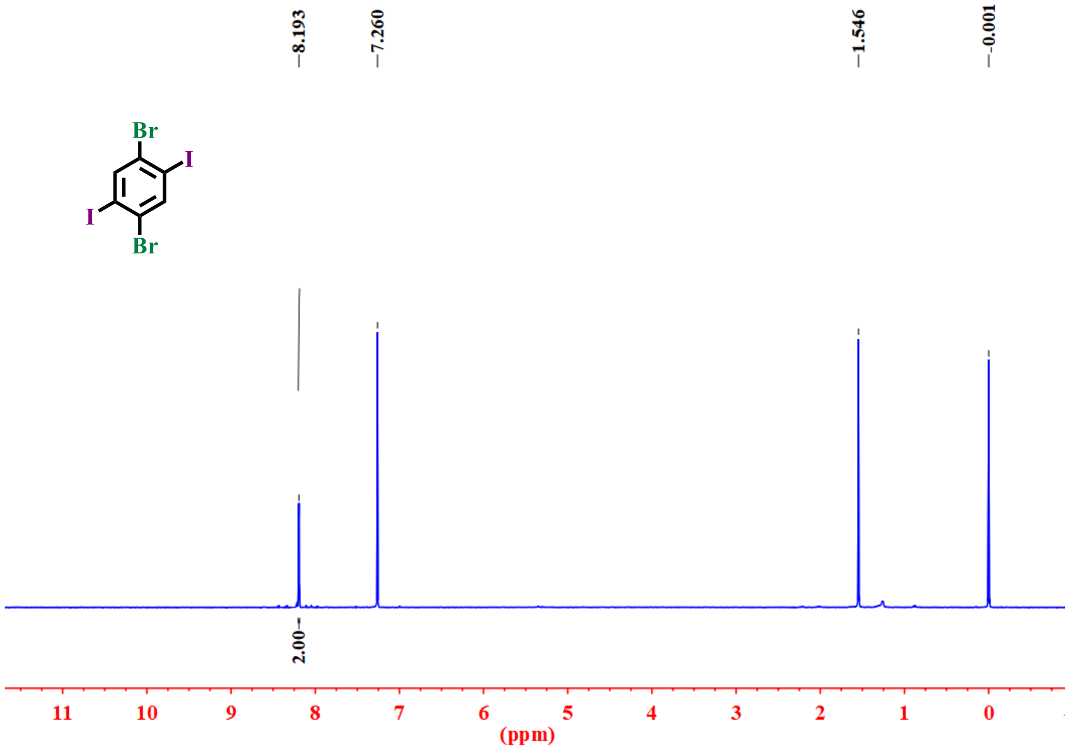

Supplementary Figure 1. ^{1}H NMR spectrum of compound **1** (400 MHz, $CDCl_3$).

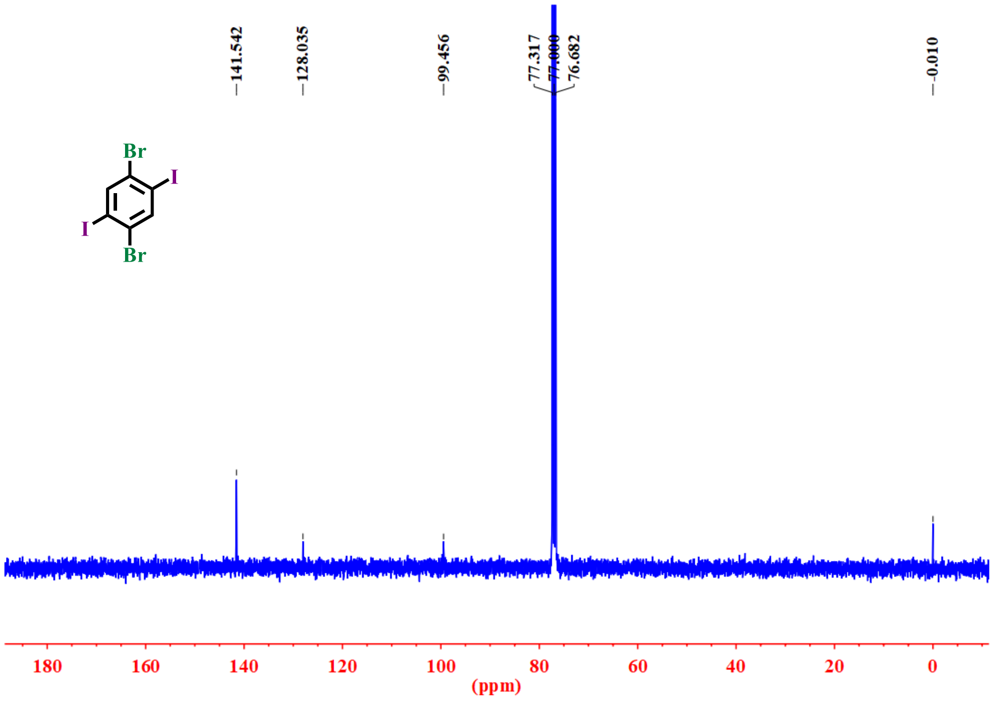

Supplementary Figure 2. ^{13}C NMR spectrum of compound **1** (100 MHz, $CDCl_3$).

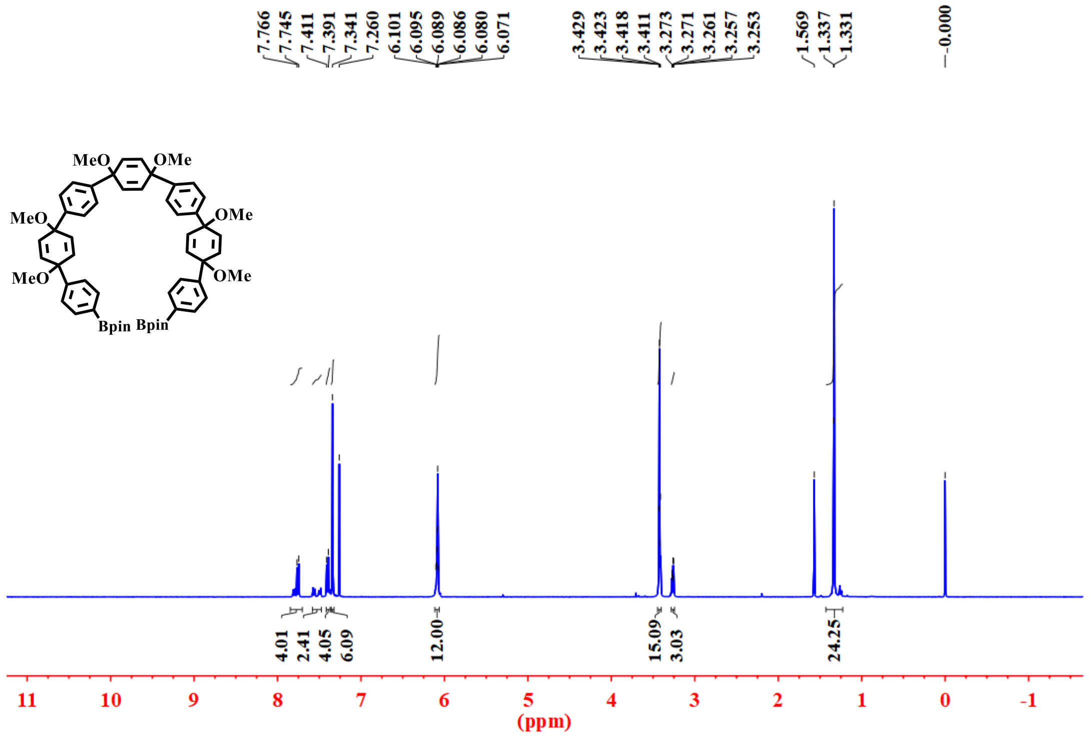

Supplementary Figure 3. ^{1}H NMR spectrum of compound **2** (400 MHz, $CDCl_3$).

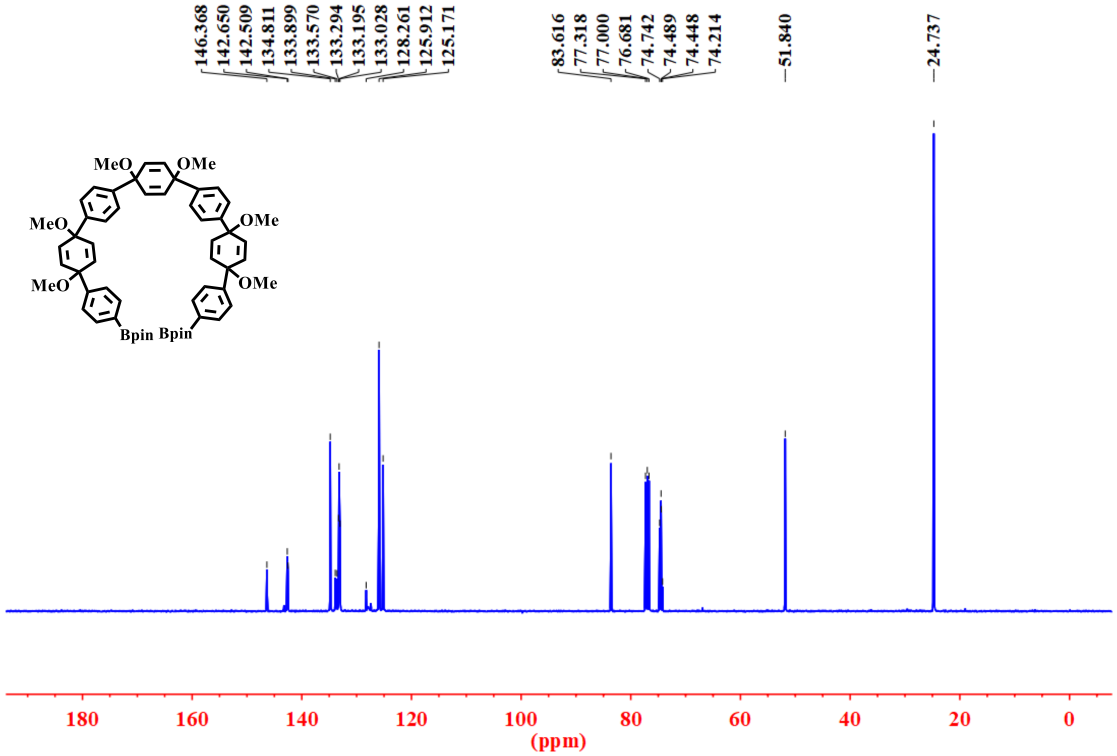

Supplementary Figure 4. ^{13}C NMR spectrum of compound **2** (100 MHz, $CDCl_3$).

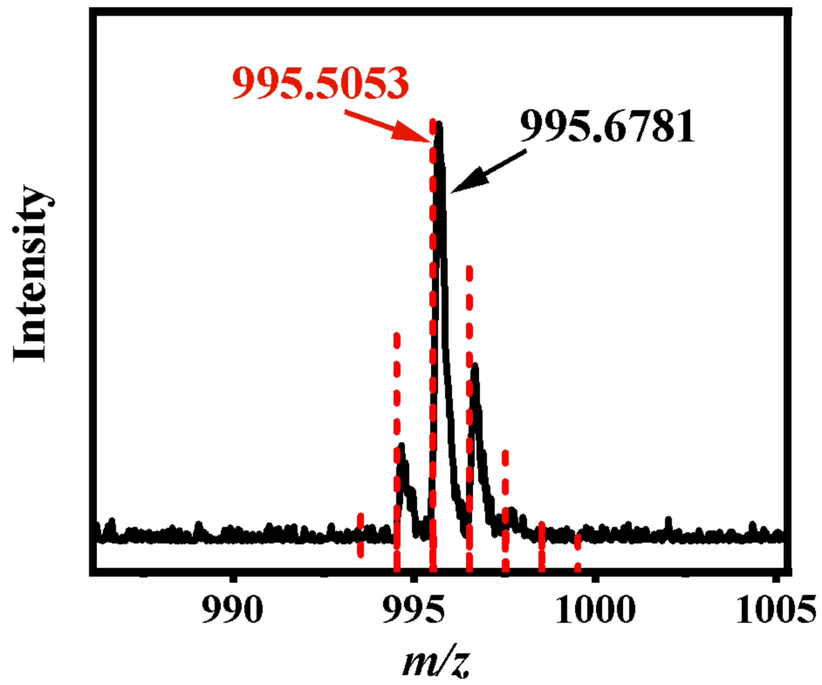

Supplementary Figure 5. MALDI-TOF-MS spectrum (black solid line) and simulated data (red dash line) for compound **2**.

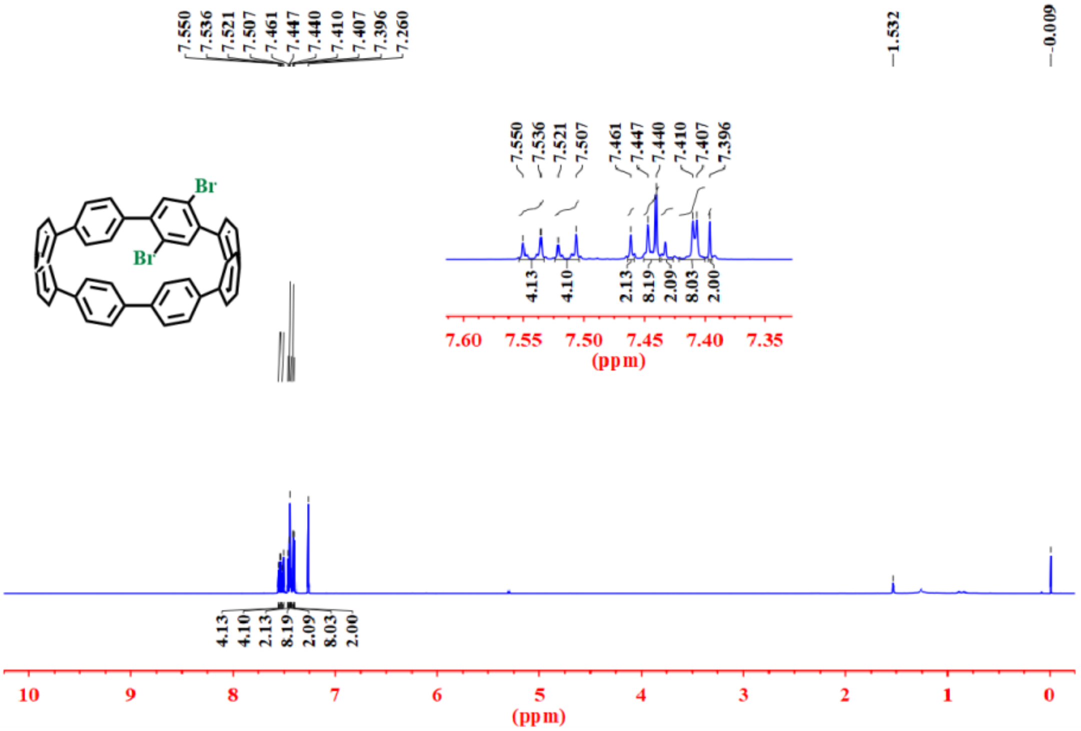

Supplementary Figure 6. ^{1}H NMR spectrum of compound **3** (600 MHz, $CDCl_3$).

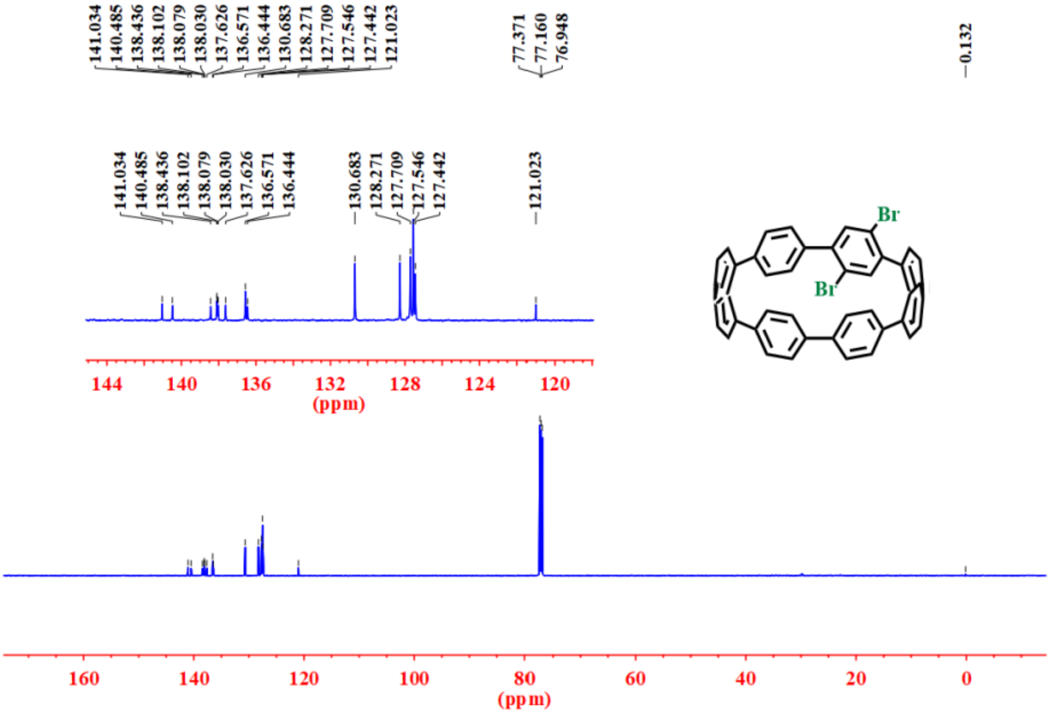

Supplementary Figure 7. ^{13}C NMR spectrum of compound **3** (150 MHz, $CDCl_3$).

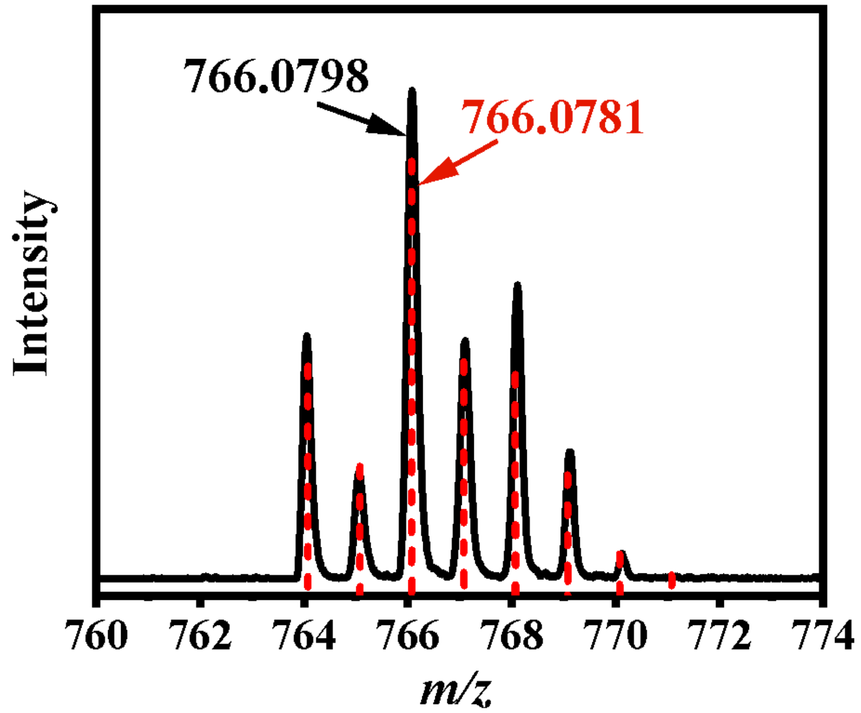

Supplementary Figure 8. MALDI-TOF-MS spectrum (black solid line) and simulated data (red dash line) for compound **3**.

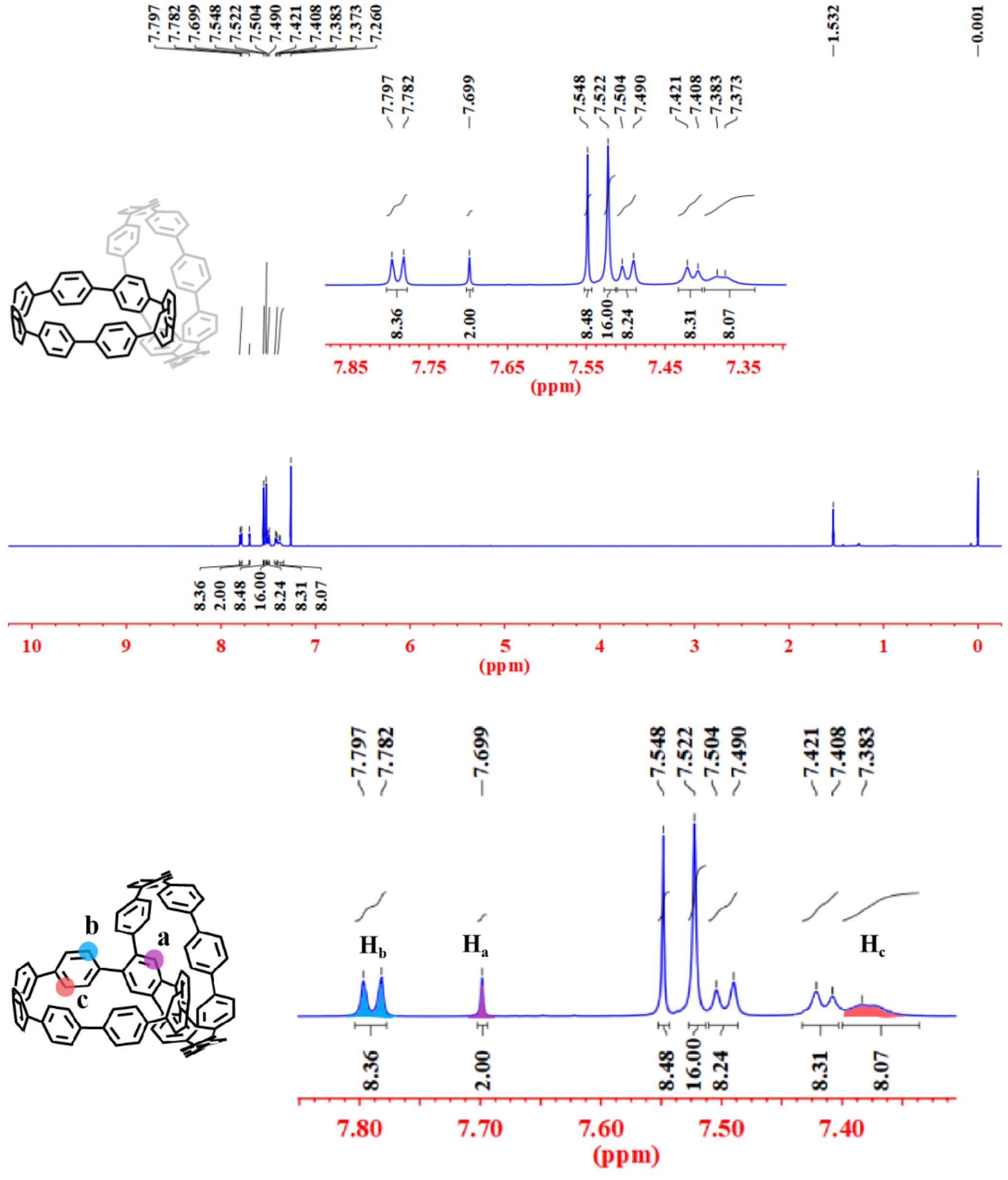

Supplementary Figure 9. ^{1}H NMR spectrum of **SCPP[8]** (600 MHz, $CDCl_3$).

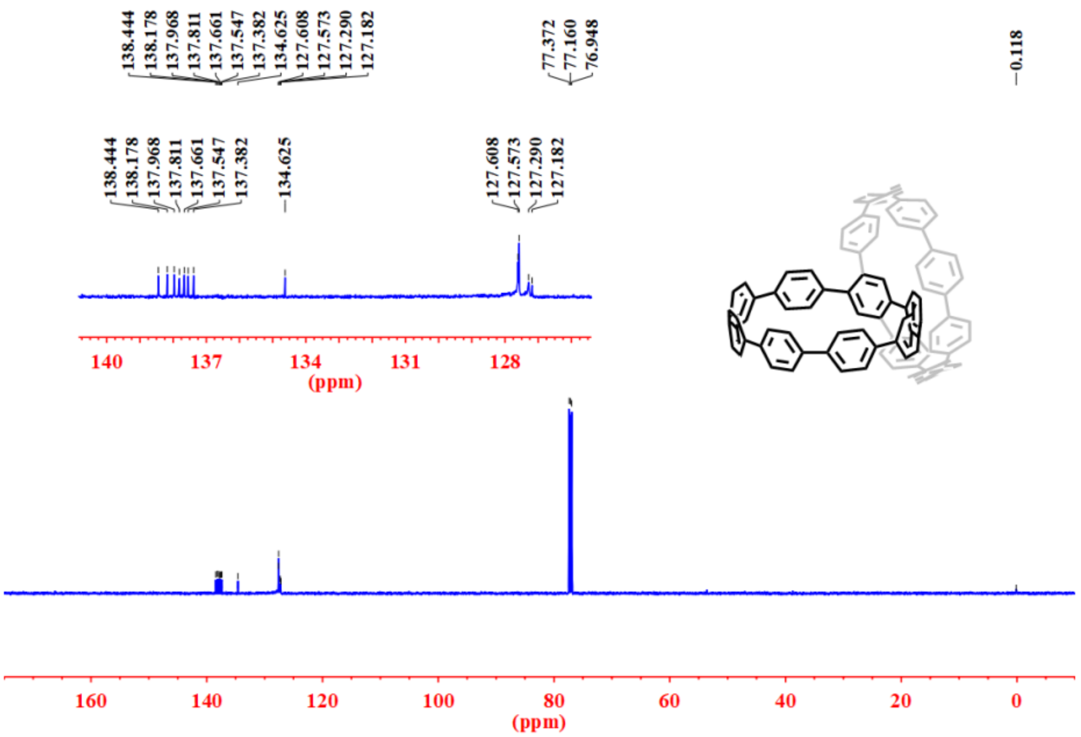

Supplementary Figure 10. ^{13}C NMR spectrum of **SCPP[8]** (150 MHz, $CDCl_3$).

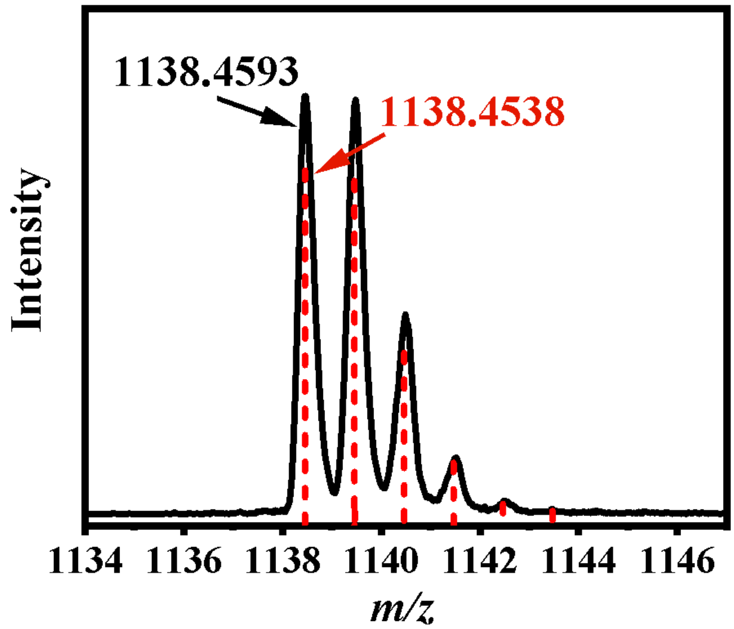

Supplementary Figure 11. MALDI-TOF-MS spectrum (black solid line) and simulated data (red dash line) for **SCPP[8]**.

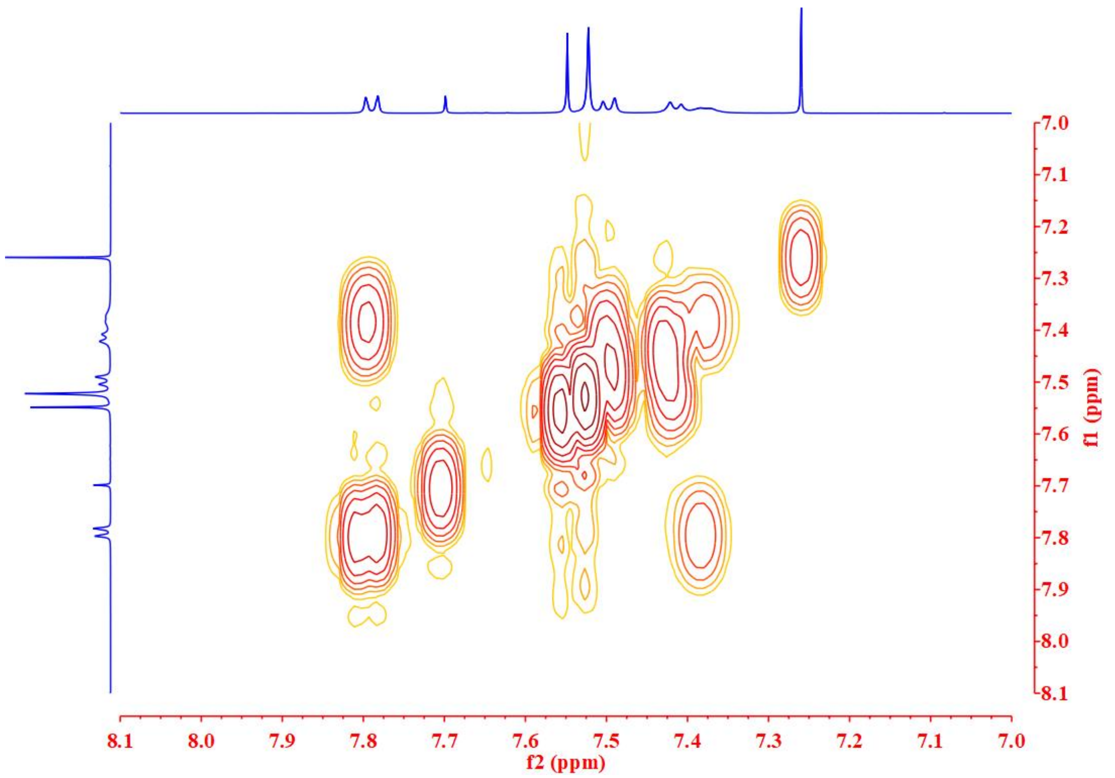

Supplementary Figure 12. Expanded 2D ^{1}H-^{1}H COSY NMR spectrum (600 MHz, $CDCl_3$) of **SCPP[8]**.

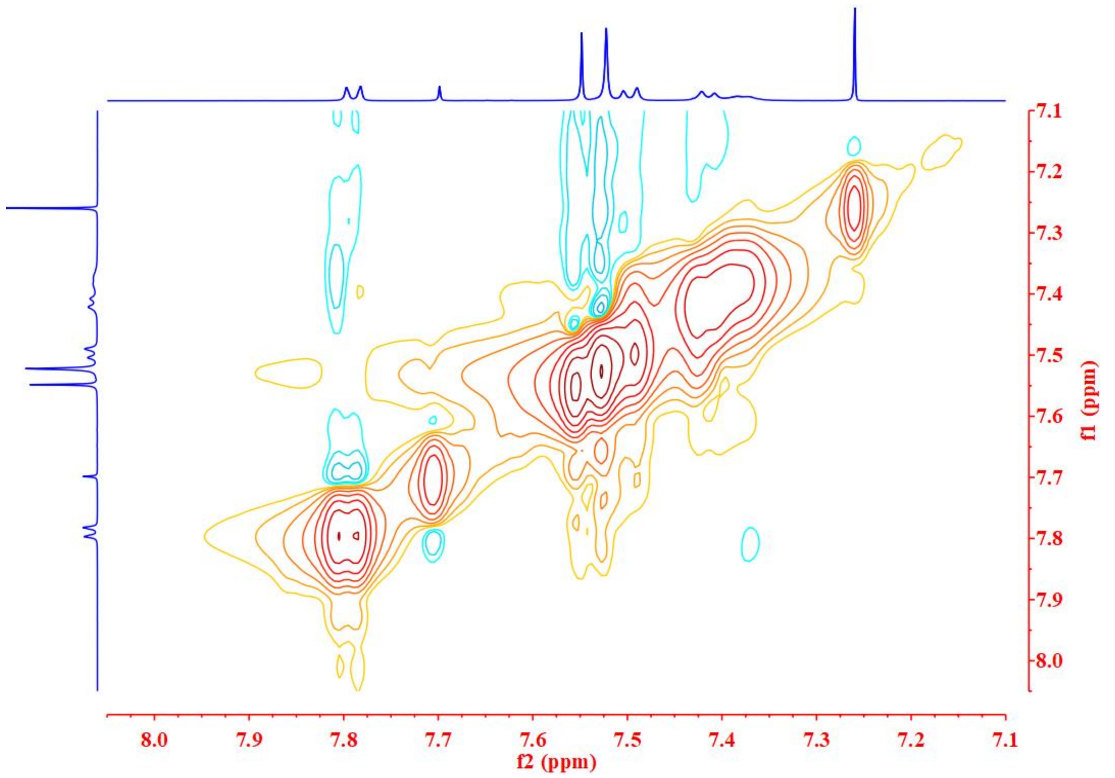

Supplementary Figure 13. Expanded 2D ^{1}H-^{1}H NOESY NMR spectrum (600 MHz, $CDCl_3$) of **SCPP[8]**.

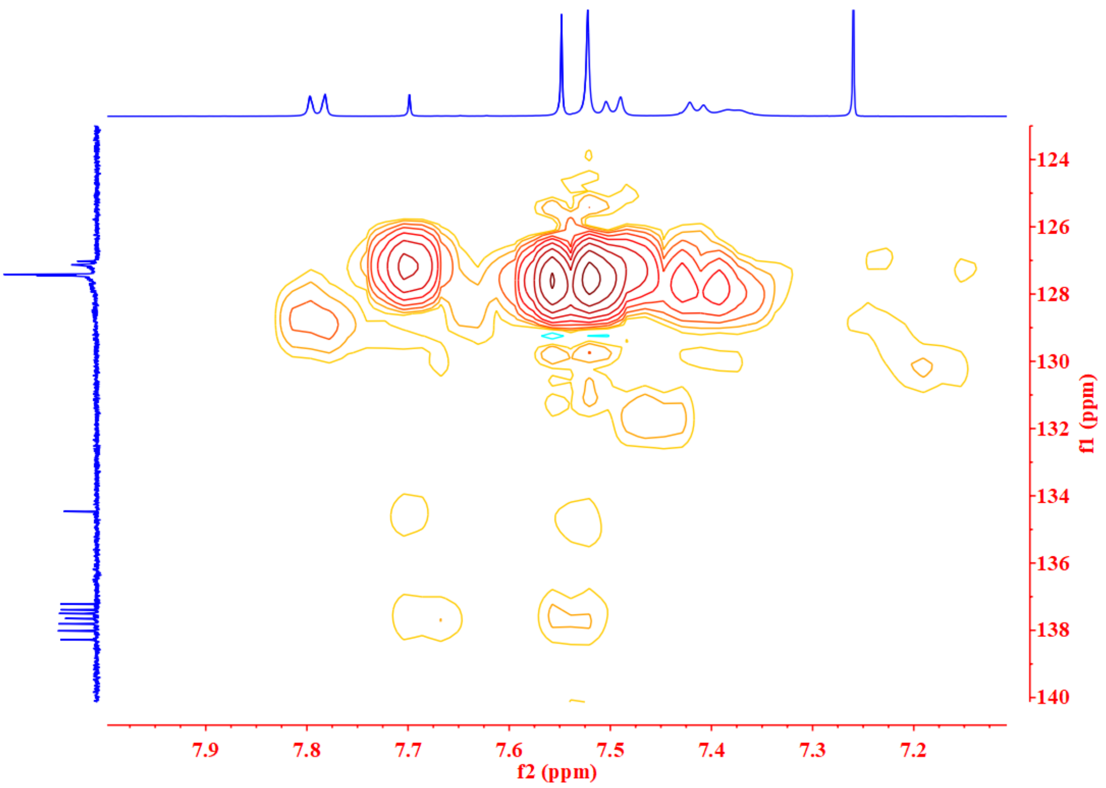

Supplementary Figure 14. Expanded 2D (H, C)-HSQC NMR spectrum (600 MHz, $CDCl_3$) of **SCPP[8]**.

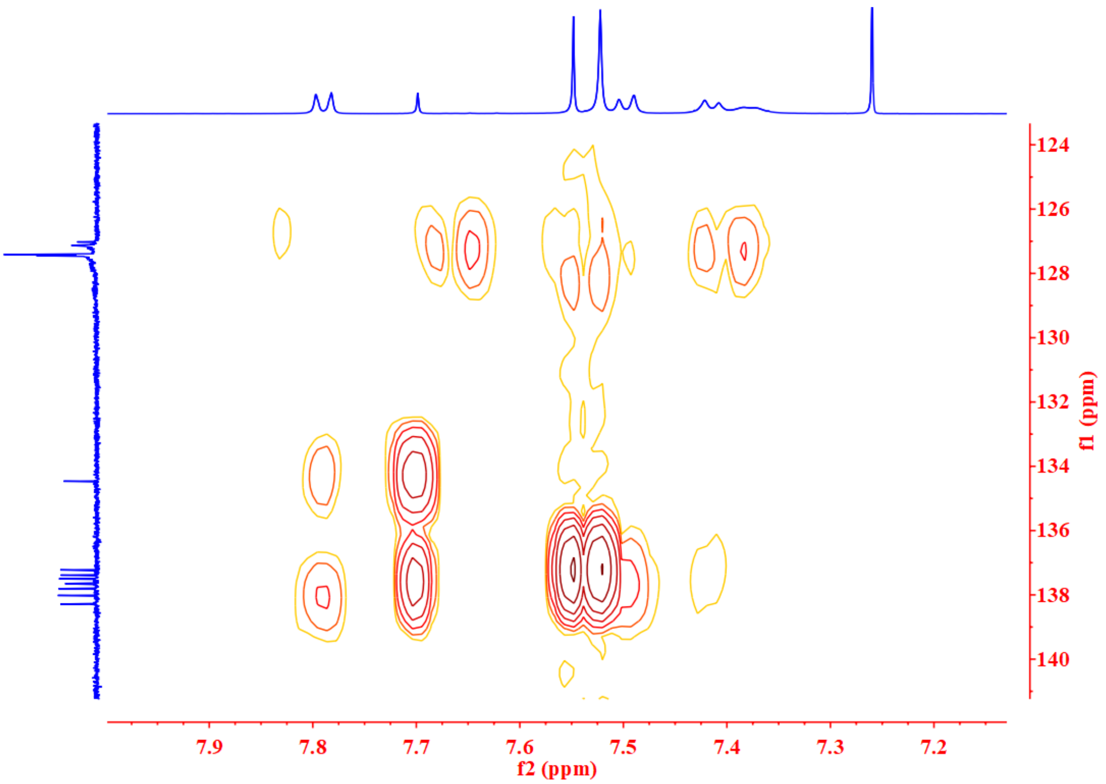

Supplementary Figure 15. Expanded 2D (H, C)-HMBC NMR spectrum (600 MHz, $CDCl_3$) of **SCPP[8]**.

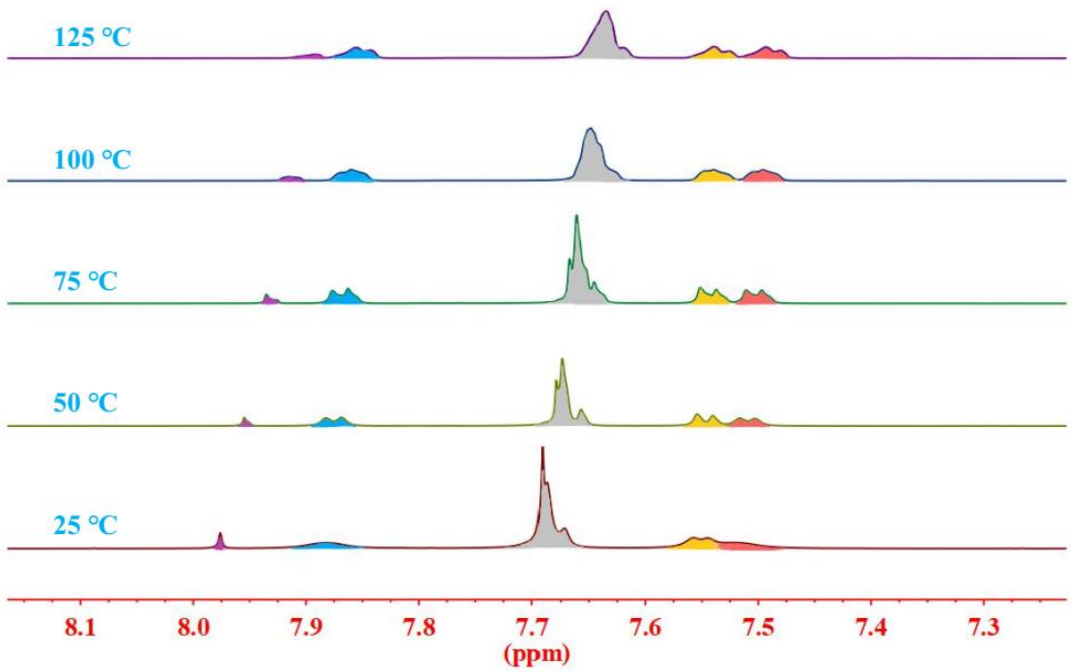

Supplementary Figure 16. The aromatic region of variable-temperature ^{1}H NMR spectra of **SCPP[8]** (600 MHz, DMSO-d_6).

Preparation of aggregates for AIE effect

Solutions with different water contents were prepared by adding THF solution of **SCPP[8]** (0.3 mL, 3 × 10^{-5} M), [8]CPP (0.3 mL, 3 × 10^{-5} M) and **SCPP[10]** (0.3 mL, 3 × 10^{-6} M) to premixed THF/H_2O (2.7 mL, with appropriate ratios), respectively. The suspensions/solutions were sonicated for 5 min before their fluorescent properties were determined.

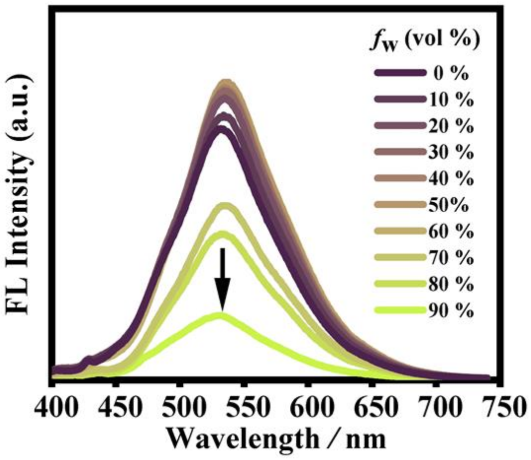

Supplementary Figure 17. Fluorescence spectra of [8]CPP in THF/H_2O mixtures with different volume fractions of H_2O (f_w).

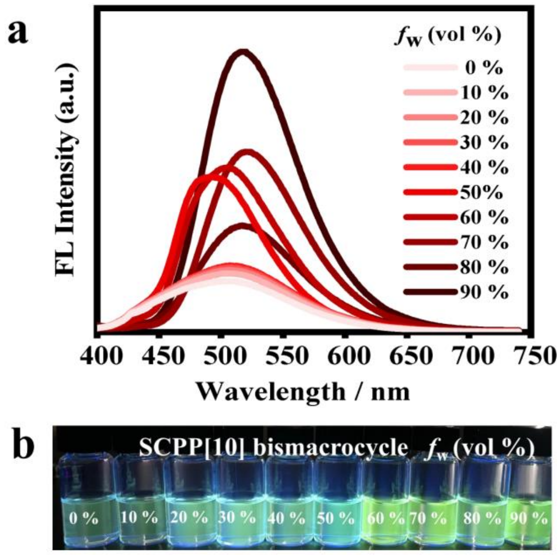

Supplementary Figure 18. AIE properties of **SCPP[10]**. **a** Fluorescence spectra of **SCPP[10]** in THF/H_2O mixtures with different volume fractions of H_2O (f_w). **b** Emission change of **SCPP[10]** in aqueous THF with f_w = 0-90 vol % under 365 nm UV light.

HPLC experiments of SCPP[8]

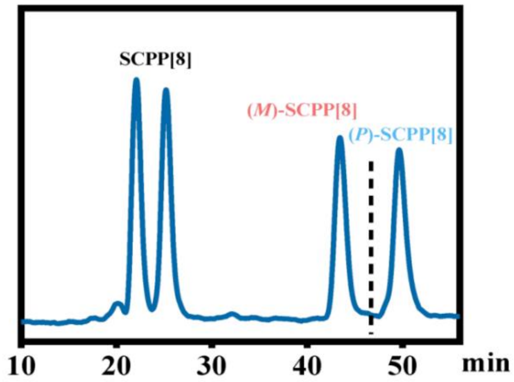

Supplementary Figure 19. Successful separation of **SCPP[8]** enantiomers.

Temperature-dependent CD spectra (*M*)/(P)-SCPP[8]

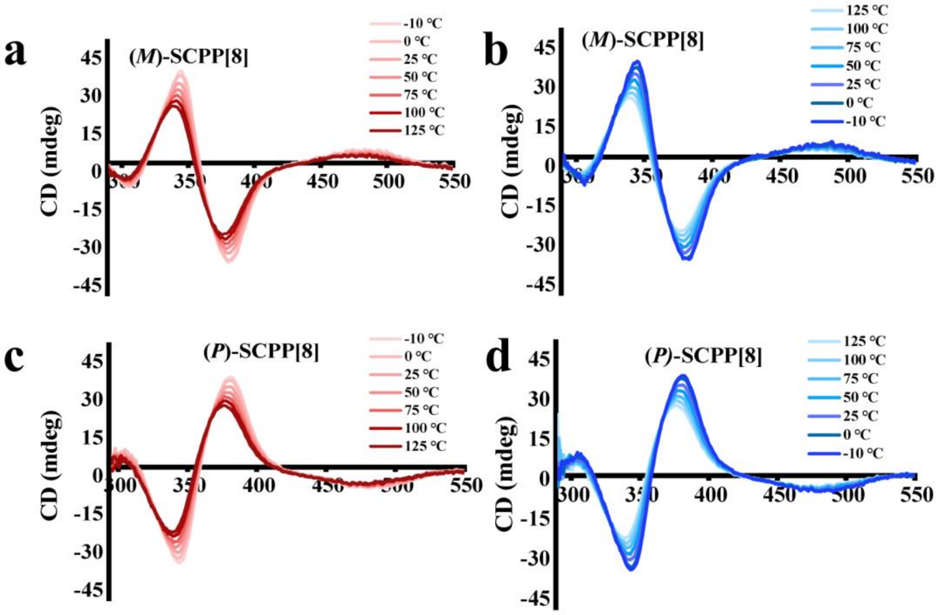

Supplementary Figure 20. Temperature-dependent CD of **(*M*)/(*P*)-SCPP[8]**. **a** Temperature-dependent CD spectra of **(*M*)-SCPP[8]** from -10 ℃ to 125 ℃. **b** Temperature-dependent CD spectra of **(*M*)-SCPP[8]** from 125 ℃ to -10 ℃. **c** Temperature-dependent CD spectra of **(*P*)-SCPP[8]** from -10 ℃ to 125 ℃. **d** Temperature-dependent CD spectra of **(*P*)-SCPP[8]** from 125 ℃ to -10 ℃.

Comparison of CPL performance

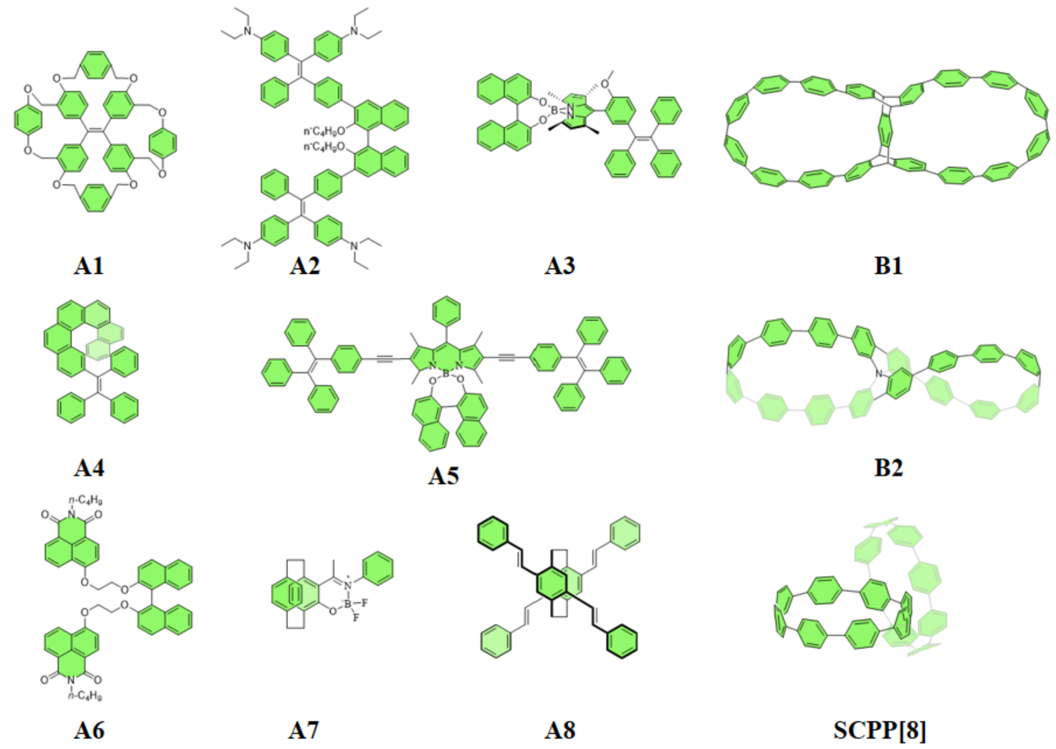

Supplementary Figure 21. The structures of **SCPP[8]** and relevant SOMs.

Supplementary Table 1. The luminescence dissymmetry factors of **SCPP[8]** and relevant SOMs

	$g_{lum}/10^{-3}$ (in solution)	$g_{lum}/10^{-3}$ (in aggregation)
(*M*)-SCPP[8]	+6.9	+19
(*M*)-A1[1]	+3.1	+6.2
(*S*)-A2[2]	-	+2.8
(*S*)-A3[3]	+4.1	-2.8
(*M*)-A4[4]	-1.1	-15
(*S*)-A5[5]	+2	+2
(*S*)-A6[6]	+5.5	-2.2
(*S*)-A7[7]	+4.5	+6.2
(*S*)-A8[8]	+3.7	+4.3
(*M*)-B1[9]	-3.5	-
(*M*)-B2[10]	-3.7	-

Computational detail

Density functional theory calculations were used to identify the performed by using Gaussian 09 software[11]. Geometrical optimization were carried out at the theoretical level of B3LYP/6-31G(d,p), where DFT-D3(BJ)[11] and Polarizable continuum model (PCM)[12] methodologies can be used to correct dispersion force and solvent effect. The resultant structures were further validated by frequency analysis without imaginary frequency. The strain energy was calculated using the computational methods reported by K. Itami[14,15,16].

SE = E(**SCPP[8]**) + 16E(Biphenyl) - E(Tetraphenyl-benzene) - 14E(Triphenyl). Moreover, time-dependent density functional theory (TD-DFT) with PBE0-1/3/6-31G(d,p)//PCM, where PBE0-1/3 features the 1/3 HF term more than pristine PBE0, were used to simulate the CD spectra.

Supplementary Table 2. Energy and Strain energy for **SCPP[8]** and [8]CPP

	**Energy(a.u.)**		**Energy(a.u.)**
**SCPP[8]**	-3464.95078363	[8]CPP	-1848.6113304
**Tetraphenyl-benzene**	-1156.64050300		-
**Biphenyl**		-463.36737140	
**Triphenyl**		-694.45656700	
**strain energy (kcal/mol)**	127.83		64.15

Supplementary Table 3. Energy level for **SCPP[8]** and [8]CPP

Name	**SCPP[8]**	[8]CPP
LUMO+3	-1.411	-0.602
LUMO+2	-1.452	-1.297
LUMO+1	-1.800	-1.298
LUMO	-2.137	-1.933
HOMO	-4.917	-5.052

HOMO-1	-5.169	-5.667
HOMO-2	-5.529	-5.667
HOMO-3	-5.542	-6.680
Gap	2.780	3.118

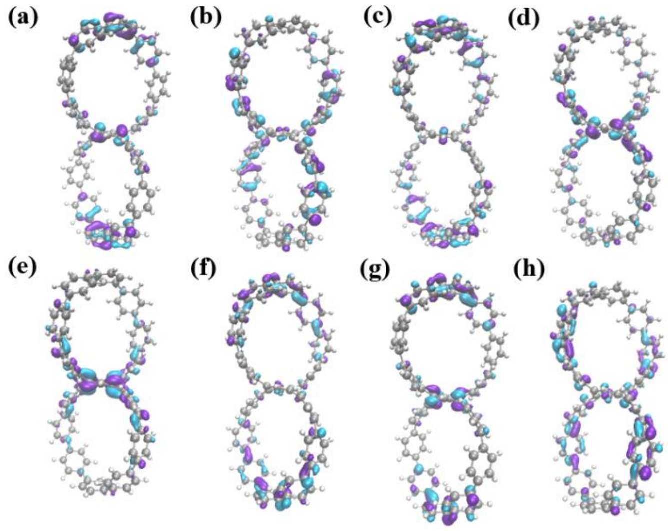

Supplementary Figure 22. Frontier molecular orbitals of **SCPP[8]**: HOMO-3(a), HOMO-2(b), HOMO-1(c), HOMO(d), LUMO(e), LUMO+1(f), LUMO+2(g) and LUMO+3(h).

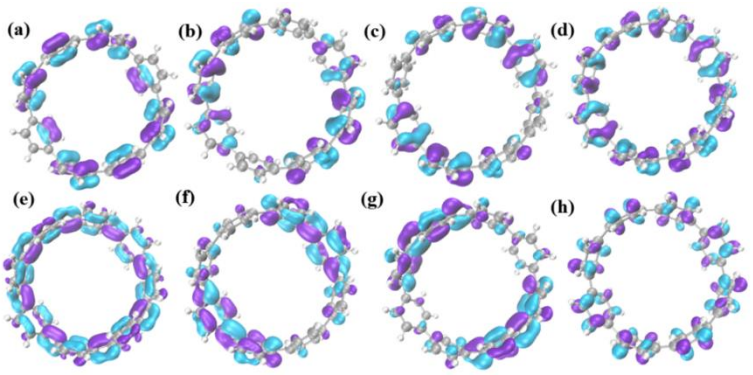

Supplementary Figure 23. Frontier molecular orbitals of [8]CPP: HOMO-3(a), HOMO-2(b), HOMO-1(c), HOMO(d), LUMO(e), LUMO+1(f), LUMO+2(g) and LUMO+3(h).

Supplementary Table 4. Oscillator strengths and transitions of the peaks from experiment and DFT calculations under the theoretical level of PBE0-1/3/6-31G(d,p)//PCM)

$\lambda_{Exp.}$	λ_{DFT}	f_{osc}	**Transitions**
**SCPP[8]**			
368	371.77	3.3466	HOMO-2→LUMO(56%)<br>HOMO-4→LUMO+1(14%)

346	340.07	0.2228	HOMO→LUMO+2(57%)<br>HOMO-2→LUMO(37%)
	333.76	1.5938	HOMO-5→LUMO(48%)
	325.57	0.4180	HOMO→LUMO+4(44%)
305	301.44	0.7219	HOMO-1→LUMO+5(50%)
	292.55	0.2244	HOMO-3→LUMO+1(44%)
	287.66	0.1531	HOMO-2→LUMO+3(50%)
[8]CPP			
340	339.69	1.8662	HOMO-1→LUMO(51%)<br>HOMO→LUMO+1(45%)

Supplementary Table 5. Ground-state structure of **SCPP[8]**

**SCPP[8]**							
**C**	3.112894	1.773448	-0.615729	C	-3.174965	-2.003775	-3.376317
**C**	2.334512	0.883658	-1.379802	C	-2.469229	-0.962256	-2.779064
**C**	3.174965	2.003775	-3.376317	C	-3.811041	-2.811850	-1.211370
**C**	2.469229	0.962256	-2.779064	C	-4.067002	-4.372321	-3.129657
**C**	3.811041	2.811850	-1.211370	C	-3.775740	-3.016382	-2.603712
**C**	4.067002	4.372321	-3.129657	C	-3.344605	-4.871961	-4.229605
**C**	3.775740	3.016382	-2.603712	C	-4.613206	-6.675341	-2.554516
**C**	3.344605	4.871961	-4.229605	C	-4.805526	-5.309533	-2.382881
**C**	4.613206	6.675341	-2.554516	C	-3.697749	-7.169480	-3.502484
**C**	4.805526	5.309533	-2.382881	C	-3.177535	-6.238389	-4.421168
**C**	3.697749	7.169480	-3.502484	C	-3.634935	-9.508435	-2.502269
**C**	3.177535	6.238389	-4.421168	C	-3.064561	-8.495377	-3.297322
**C**	3.634935	9.508435	-2.502269	C	-0.918189	-9.614342	-3.027265
**C**	3.064561	8.495377	-3.297322	C	-1.706313	-8.645934	-3.630564
**C**	0.918189	9.614342	-3.027265	C	-2.843517	-10.481398	-1.896817
**C**	1.706313	8.645934	-3.630564	C	-1.444037	-10.486593	-2.056777
**C**	2.843517	10.481398	-1.896817	H	3.061235	1.731600	0.465838
**C**	0.514491	11.073539	-1.060061	H	3.194368	2.066019	-4.459763
**C**	1.444037	10.486593	-2.056777	H	1.958685	0.245150	-3.414290
**C**	-0.867591	11.169858	-1.309982	H	4.272239	3.552019	-0.568125
**C**	0.000000	11.198402	1.318754	H	2.799634	4.189666	-4.873095
**C**	0.922490	11.217846	0.279337	H	5.085817	7.356128	-1.854635
**C**	-1.370964	10.998838	1.068416	H	5.456072	4.973689	-1.582376
**C**	-1.788822	11.116309	-0.270620	H	2.540710	6.574987	-5.232442
**C**	-1.662743	9.509563	3.033087	H	4.698592	9.492677	-2.286161
**C**	-2.253318	10.340259	2.063352	H	-0.150179	9.585263	-3.205841
**C**	-4.362758	9.195528	2.505514	H	1.217467	7.905465	-4.252698
**C**	-3.648133	10.227468	1.902177	H	3.314919	11.193784	-1.227074
**C**	-2.374356	8.482458	3.634319	H	-1.236204	11.166280	-2.330261
**C**	-4.245652	6.857104	3.502497	H	0.368292	11.219318	2.338956

**C**	-3.716726	8.228245	3.299684	H	1.978652	11.210493	0.526433
**C**	-5.118322	6.294484	2.552377	H	-2.841485	11.030518	-0.517902
**C**	-3.716429	4.593064	4.227882	H	-0.595495	9.562502	3.212629
**C**	-3.656349	5.968113	4.421181	H	-5.421896	9.098239	2.288489
**C**	-4.395772	4.040108	3.126010	H	-4.172571	10.902338	1.233051
**C**	-5.203578	4.918059	2.378940	H	-1.830403	7.780917	4.255818
**C**	-3.320876	1.748708	3.370949	H	-5.641185	6.937362	1.852383
**C**	-3.999372	2.711469	2.599014	H	-3.121518	3.954447	4.871958
**C**	-3.242487	1.524523	0.610334	H	-3.049148	6.352138	5.234109
**C**	-4.019291	2.505109	1.206628	H	-5.824666	4.533681	1.576916
**C**	-2.536096	0.765773	2.773066	H	-3.344394	1.808873	4.454391
**C**	-2.396336	0.698253	1.373730	H	-3.188569	1.486835	-0.471275
**C**	-0.514491	-11.073539	-1.060061	H	-4.537221	3.207037	0.563698
**C**	0.867591	-11.169858	-1.309982	H	-1.970528	0.090908	3.407967
**C**	0.000000	-11.198402	1.318754	H	1.236204	-11.166280	-2.330261
**C**	-0.922490	-11.217846	0.279337	H	-0.368292	-11.219318	2.338956
**C**	1.370964	-10.998838	1.068416	H	-1.978652	-11.210493	0.526433
**C**	1.788822	-11.116309	-0.270620	H	2.841485	-11.030518	-0.517902
**C**	1.662743	-9.509563	3.033087	H	0.595495	-9.562502	3.212629
**C**	2.253318	-10.340259	2.063352	H	5.421896	-9.098239	2.288489
**C**	4.362758	-9.195528	2.505514	H	4.172571	-10.902338	1.233051
**C**	3.648133	-10.227468	1.902177	H	1.830403	-7.780917	4.255818
**C**	2.374356	-8.482458	3.634319	H	5.641185	-6.937362	1.852383
**C**	4.245652	-6.857104	3.502497	H	3.121518	-3.954447	4.871958
**C**	3.716726	-8.228245	3.299684	H	3.049148	-6.352138	5.234109
**C**	5.118322	-6.294484	2.552377	H	5.824666	-4.533681	1.576916
**C**	3.716429	-4.593064	4.227882	H	3.344394	-1.808873	4.454391
**C**	3.656349	-5.968113	4.421181	H	3.188569	-1.486835	-0.471275
**C**	4.395772	-4.040108	3.126010	H	4.537221	-3.207037	0.563698
**C**	5.203578	-4.918059	2.378940	H	1.970528	-0.090908	3.407967
**C**	3.320876	-1.748708	3.370949	H	0.000000	0.000000	2.448119
**C**	3.999372	-2.711469	2.599014	H	0.000000	0.000000	-2.454745
**C**	3.242487	-1.524523	0.610334	H	-3.061235	-1.731600	0.465838
**C**	4.019291	-2.505109	1.206628	H	-3.194368	-2.066019	-4.459763
**C**	2.536096	-0.765773	2.773066	H	-1.958685	-0.245150	-3.414290
**C**	1.227090	-0.094184	0.694560	H	-4.272239	-3.552019	-0.568125
**C**	2.396336	-0.698253	1.373730	H	-2.799634	-4.189666	-4.873095
**C**	1.215991	0.189696	-0.701092	H	-5.085817	-7.356128	-1.854635
**C**	-1.227090	0.094184	0.694560	H	-5.456072	-4.973689	-1.582376
**C**	0.000000	0.000000	1.363402	H	-2.540710	-6.574987	-5.232442
**C**	-1.215991	-0.189696	-0.701092	H	-4.698592	-9.492677	-2.286161
**C**	0.000000	0.000000	-1.369994	H	0.150179	-9.585263	-3.205841
**C**	-3.112894	-1.773448	-0.615729	H	-1.217467	-7.905465	-4.252698
**C**	-2.334512	-0.883658	-1.379802	H	-3.314919	-11.193784	-1.227074

Supplementary Table 6. Ground-state structure of [8]CPP

[8]CPP							
**C**	4.502628	1.846310	-1.546478	C	3.636553	-4.800678	-0.545728
**C**	5.288466	1.460380	-0.445403	C	3.918468	-3.844129	0.448974
**C**	4.797192	3.635448	0.546291	C	5.456035	-0.910597	-1.293350
**C**	5.504040	2.435254	0.547951	C	5.544037	0.013862	-0.235511
**C**	3.798839	3.041157	-1.548173	C	4.842772	-2.702136	0.241020
**C**	2.700048	4.843439	-0.241435	C	5.097431	-2.233503	-1.061419
**C**	3.841263	3.917870	-0.448762	C	5.208894	-1.849249	1.299537
**C**	2.232355	5.100325	1.060943	C	5.535586	-0.518239	1.067557
**C**	0.516614	5.537544	-1.067848	H	4.313251	1.142153	-2.348094
**C**	1.847297	5.209631	-1.300026	H	4.935294	4.322939	1.374933
**C**	-0.014962	5.547498	0.235389	H	6.172470	2.225169	1.377054
**C**	0.909846	5.460274	1.292993	H	3.092788	3.215729	-2.351437
**C**	-2.436563	5.507800	-0.547393	H	2.868165	4.890765	1.914463
**C**	-1.461427	5.292011	0.445710	H	-0.142734	5.657110	-1.920863
**C**	-3.041775	3.801789	1.548269	H	2.190097	5.126770	-2.325995
**C**	-1.847297	4.506306	1.546884	H	0.571691	5.560205	2.318993
**C**	-3.636553	4.800678	-0.545728	H	-2.226530	6.176219	-1.376533
**C**	-4.842772	2.702136	0.241020	H	-3.215490	3.094875	2.350996
**C**	-3.918468	3.844129	0.448974	H	-1.143433	4.317681	2.348920
**C**	-5.208894	1.849249	1.299537	H	-4.324183	4.938851	-1.374238
**C**	-5.456035	0.910597	-1.293350	H	-5.126856	2.192458	2.325450
**C**	-5.097431	2.233503	-1.061419	H	-5.554805	0.572082	-2.319325
**C**	-5.544037	-0.013862	-0.235511	H	-4.887024	2.868848	-1.915070
**C**	-5.535586	0.518239	1.067557	H	-5.655491	-0.140801	1.920788
**C**	-4.502628	-1.846310	-1.546478	H	-4.313251	-1.142153	-2.348094
**C**	-5.288466	-1.460380	-0.445403	H	-4.935294	-4.322939	1.374933
**C**	-4.797192	-3.635448	0.546291	H	-6.172470	-2.225169	1.377054

**C**	-5.504040	-2.435254	0.547951	H	-3.092788	-3.215729	-2.351437
**C**	-3.798839	-3.041157	-1.548173	H	-2.868165	-4.890765	1.914463
**C**	-2.700048	-4.843439	-0.241435	H	0.142734	-5.657110	-1.920863
**C**	-3.841263	-3.917870	-0.448762	H	-2.190097	-5.126770	-2.325995
**C**	-2.232355	-5.100325	1.060943	H	-0.571691	-5.560205	2.318993
**C**	-0.516614	-5.537544	-1.067848	H	2.226530	-6.176219	-1.376533
**C**	-1.847297	-5.209631	-1.300026	H	3.215490	-3.094875	2.350996
**C**	0.014962	-5.547498	0.235389	H	1.143433	-4.317681	2.348920
**C**	-0.909846	-5.460274	1.292993	H	4.324183	-4.938851	-1.374238
**C**	2.436563	-5.507800	-0.547393	H	4.887024	-2.868848	-1.915070
**C**	1.461427	-5.292011	0.445710	H	5.655491	0.140801	1.920788
**C**	3.041775	-3.801789	1.548269	H	5.554805	-0.572082	-2.319325
**C**	1.847297	-4.506306	1.546884	H	5.126856	-2.192458	2.325450

Supplementary References

1. Xiong, J.-B. *et al.* The fixed propeller-like conformation of tetraphenylethylene that reveals aggregation-induced emission effect, chiral recognition, and enhanced chiroptical property. *J. Am. Chem. Soc.* **138**, 11469-11472 (2016).
2. Zhang, X. *et al.* High brightness circularly polarized organic light-emitting diodes based on nondoped aggregation-induced emission (AIE)-active chiral binaphthyl emitters. *Org. Lett.* **21**, 439-443 (2018).
3. Feng, H.-T., Gu, X., Lam, J. W. Y., Zheng, Y.-S. & Tang, B. Z. Design of multi-functional AIEgens: tunable emission, circularly polarized luminescence and self-assembly by dark through-bond energy transfer. *J. Mater. Chem. C* **6**, 8934-8940 (2018).
4. Shen, C. *et al.* Helicene-derived aggregation-induced emission conjugates with highly tunable circularly polarized luminescence. *Mater. Chem. Front.* **4**, 837-844 (2020).
5. Zhang, S. *et al.* Circularly polarized luminescence of AIE-active chiral O-BODIPYs induced via intramolecular energy transfer. *Chem. Commun.* **51**, 9014-9017 (2015).
6. Sheng, Y. *et al.* Reversal circularly polarized luminescence of AIE-active chiral binaphthyl molecules from solution to aggregation. *Chem. Eur. J.* **21**, 13196-13200 (2015).

7. Li, K. *et al.* 3D boranil complexes with aggregation-amplified circularly polarized luminescence. *J. Org. Chem.* **86**, 16707-16715 (2021).
8. Gon, M., Morisaki, Y. & Chujo, Y. Optically active phenylethene dimers based on planar chiral tetrasubstituted [2.2]paracyclophane. *Chem. Eur. J.* **23**, 6323-6329 (2017).
9. Xu, W. *et al.* Synthesis and characterization of a pentiptycene-derived dual oligoparaphenylene nanohoop. *Angew. Chem. Int. Ed.* **58**, 3943-3947 (2019).
10. Senthilkumar, K. *et al.* Lemniscular [16]cycloparaphenylene: a radially conjugated figure-eight aromatic molecule. *J. Am. Chem. Soc.* **141**, 7421-7427 (2019).
11. Frisch M*, et al.* Gaussian 09, Revision d. 01, Gaussian. *Inc, Wallingford CT* **201** (2009).
12. Yanai T, Tew DP, Handy NC. A new hybrid exchange-correlation functional using the Coulomb-attenuating method (CAM-B3LYP). *Chem. Phys. Lett.* **393**, 51-57 (2004).
13. Grimme S, Ehrlich S, Goerigk L. Effect of the damping function in dispersion corrected density functional theory. *J. Comput. Chem.* **32**, 1456-1465 (2011).
14. Segawa Y, Omachi H, Itami K. Theoretical studies on the structures and strain energies of cycloparaphenylenes. *Org. Lett.* **12**, 2262-2265 (2010).
15. Takaba H, Omachi H, Yamamoto Y, Bouffard J, Itami K. Selective synthesis of [12]cycloparaphenylene. *Angew. Chem. Int. Ed.* **48**, 6112-6116 (2009).
16. Wheeler SE, Houk KN, Schleyer PvR, Allen WD. A Hierarchy of homodesmotic reactions for thermochemistry. *J. Am. Chem. Soc.* **131**, 2547-2560 (2009).
